# A haplotype resolved chromosomal level avocado genome allows analysis of novel avocado genes

**DOI:** 10.1093/hr/uhac157

**Published:** 2022-08-01

**Authors:** Onkar Nath, Stephen J Fletcher, Alice Hayward, Lindsay M Shaw, Ardashir Kharabian Masouleh, Agnelo Furtado, Robert J Henry, Neena Mitter

**Affiliations:** Queensland Alliance for Agriculture and Food Innovation, The University of Queensland, Brisbane 4072 Australia; Queensland Alliance for Agriculture and Food Innovation, The University of Queensland, Brisbane 4072 Australia; Queensland Alliance for Agriculture and Food Innovation, The University of Queensland, Brisbane 4072 Australia; Queensland Alliance for Agriculture and Food Innovation, The University of Queensland, Brisbane 4072 Australia; Queensland Alliance for Agriculture and Food Innovation, The University of Queensland, Brisbane 4072 Australia; Queensland Alliance for Agriculture and Food Innovation, The University of Queensland, Brisbane 4072 Australia; Queensland Alliance for Agriculture and Food Innovation, The University of Queensland, Brisbane 4072 Australia; Queensland Alliance for Agriculture and Food Innovation, The University of Queensland, Brisbane 4072 Australia

## Abstract

Avocado (*Persea americana*) is a member of the magnoliids, an early branching lineage of angiosperms that has high value globally with the fruit being highly nutritious. Here, we report a chromosome-level genome assembly for the commercial avocado cultivar Hass, which represents 80% of the world’s avocado consumption. The DNA contigs produced from Pacific Biosciences HiFi reads were further assembled using a previously published version of the genome supported by a genetic map. The total assembly was 913 Mb with a contig N50 of 84 Mb. Contigs assigned to the 12 chromosomes represented 874 Mb and covered 98.8% of benchmarked single-copy genes from embryophytes. Annotation of protein coding sequences identified 48 915 avocado genes of which 39 207 could be ascribed functions. The genome contained 62.6% repeat elements. Specific biosynthetic pathways of interest in the genome were investigated. The analysis suggested that the predominant pathway of heptose biosynthesis in avocado may be through sedoheptulose 1,7 bisphosphate rather than via alternative routes. Endoglucanase genes were high in number, consistent with avocado using cellulase for fruit ripening. The avocado genome appeared to have a limited number of translocations between homeologous chromosomes, despite having undergone multiple genome duplication events. Proteome clustering with related species permitted identification of genes unique to avocado and other members of the Lauraceae family, as well as genes unique to species diverged near or prior to the divergence of monocots and eudicots. This genome provides a tool to support future advances in the development of elite avocado varieties with higher yields and fruit quality.

## Introduction

Avocado (*Persea americana*) is a member of the flowering plant family Lauraceae, which includes at least 2500 pantropical to temperate species, most of which are woody trees [13]. This family contains a few edible fruit trees, and includes cinnamon, bay laurel and sassafras [6]. Avocado has been divided into three landraces: Mexican, Guatemalan and West Indian [46]. Hass, a hybrid of the Guatemalan and Mexican races (without a known breeding history), is the principal cultivar, originating from a chance seedling that has been clonally propagated ever since [46]. The accidental nature of this cultivar along with its subsequent commercial prominence supports the need for more deliberate efforts to achieve genetic improvement and a high-quality genome to support avocado breeding.

A limited number of members of the Lauraceae have been sequenced, including *P. americana* (avocado) [43], *Cinnamomum kanehirae* (Chinese Sassafras) [9] and *Litsea cubeba* (Mountain Pepper) [13]. These magnoliid genomes provide insights into the evolution of flowering plants as they are either the sister group to the monocots, eudicots, or to both together [7, 9, 10, 13]. This incongruence may be biological rather than methodological in basis, stemming from ancient and pervasive incomplete lineage sorting that generated genomic blocks with different ancestries among these three major angiosperm lineages [43].

**Figure 1:**
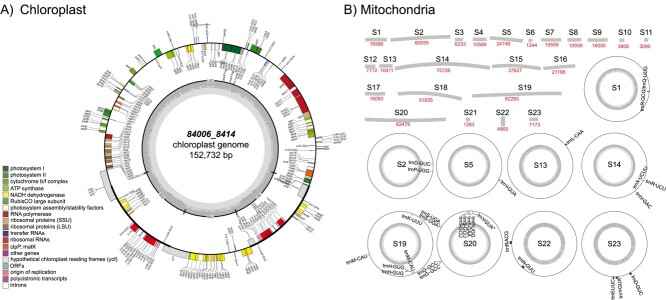
*Plastome and Mitochondrial genome maps of Persea* *americana* cultivar Hass*. A) Chloroplast genome and annotation. The SSC subunit of the plastome was found to occur in both orientations giving rise to two distinct chloroplast sequences. Genes present outside the outer track are transcribed clockwise and the ones present inside are transcribed anti-clockwise. Genes belonging to different functional groups are colour-coded. B) Mitochondrial contigs and annotation. The sizes of contigs are indicated under each contig. As plant mitochondrial genomes generally exist in small circular and branched form, the annotation was represented on circular contigs. The annotations correspond to identified tRNA sites on the mitochondrial contigs.*

To date, much of the genetic protentional of avocado is yet to be unlocked via modern breeding programmes. A high-quality genome with a complete gamut of genes will serve not only as a basis for advancing evolutionary studies, but as an important tool in the development of elite varieties with increased fruit quality and yield under biotic and abiotic stresses. Rendon-Anaya et al. [43] produced an assembly of the Hass cultivar comprising 8135 contigs, of which 915 were anchored using a genetic map, and sequenced a native Mexican avocado (*Persea americana var. drymifolia*). This first anchored avocado genome covered about half of the expected genome size, and the assembly (N50 296 371 bp) was highly fragmented. Herein, we report a new chromosome level phased genome assembly of the cultivar Hass as a resource for future genome-assisted breeding efforts.

## Results

### Genome sequencing and assembly

The Hass cultivar was sequenced and assembled to chromosome level using HiFi reads from PacBio sequencing. The input DNA was sheared, leading to an average read length of 15 kb. The three PacBio SMRT cells sequenced resulted in 267, 210 and 634 Gb of raw reads which were collapsed to obtain 39, 31 and 92 Gb circular consensus sequencing (CCS) files (Supplementary Table S2, Fig. S2). Most of the sequenced reads were in the length range of 10 kb to 25 kb as expected. The estimated genome coverage of the sequenced reads were 9.2x, 11.5x and 27.2x respectively, leading to a total of 47.9x coverage of the expected 920 Mb genome [43].

The genome was assembled using the Hifiasm tool V. 0.16.1-r375 [14], resulting in a 935 Mb genome in 1063 contigs. The N50 of the assembly was 26 Mb. Many of the smaller contigs were organellar, and the nuclear genome after removal of these contigs was 913 Mb (see below). The BUSCO tool [35] indicated the genome contained 98.9% of the benchmarked, conserved, single-copy orthologous genes from the embryophyte lineage.

Hifiasm was also used to generate haplotig assemblies. The two haplotig assemblies comprised 907 Mb in 1347 contigs and 883 Mb in 641 contigs with N50 values of 7.5 Mb and 7.3 Mb respectively. Comparison maps were constructed among the sets of two haplotigs, which indicated many regions of high similarity (Supplementary Fig. S15).

### Organellar genome assembly and annotation

The organellar genomes (i.e. mitochondrial and plastome), were assembled using the GetOrganelle [28] tool (Figure 1). The mitochondrial genome was found to be 527 406 bp in length and was assembled into 23 contigs. The mitochondrial genome of avocado is over three times the size of the chloroplast genome. The chloroplast genome comprised two assemblies of 152 732 bp each. The two assemblies varied by the SSC (Small single copy) subunit, which is in accordance with a previous discovery indicating the existence of two distinct chloroplast versions in most plants [53]. Details of the chloroplast assembly and annotation are provided in the Supplementary Appendix.

### Pseudomolecule assembly

The Hifiasm assembled contigs were screened to remove those containing organellar genomes, leaving a 913 Mb assembly in 516 contigs. Rendon-Anaya et al. [43] used SNP markers, mapping populations, microsatellite markers and 121mer probes from an Illumina chip to map scaffolds onto pseudomolecules. In the current study, contigs were mapped against the CDS regions of these 12 pseudochromosomes using SynMap [24] in CoGe [33]. This facilitated the assembly of contigs by aligning them to form pseudochromosomes. This generated 13 pseudomolecules (Table 1, Supplementary Fig. S3) of which the 12 pseudochromosomes (Chr) covered about 874 Mb, i.e. 95.0% of the predicted avocado genome size (920 Mb). Rendon-Anaya et al. [43] estimated the genome size to be 920 Mb based on their sequence-based methods, suggesting 823.4 Mb and 912.7 Mb for the Hass cultivar and var. *drymifolia*, respectively. Our assembly is larger than the previously estimated Hass genome size. A length distribution graph indicates that the 12 pseudochromosomes (Chr1 to Chr12) were comprised of longer contigs, whereas an unordered Chr0 was built from shorter contigs (Supplementary Fig. S3). Furthermore, BUSCO analysis indicated 100% completeness for the eukaryote lineage and 98.8% completeness for the embryophyte lineage. The quality value (QV) assessed using Inspector tool [12] indicated a 35.09 QV score. A higher QV suggests a more accurately assembled genome, with 35.09 corresponding to 99.9% accuracy [44].

**Table 1:** Pseudo-molecule assembly statistics. This table indicates chromosome-wise statistics of the assembled genome

# Chr	Chr size (bp)	No. of Contigs forming Chr	GC content (%)	Repeats (%)	CDS length (%)	Coding sequences (%)	CDS count	No. of ORFs
Chr1	97 805 473	5	38.25	60.54	5 571 716	5.7	4960	35 951
Chr2	103 193 853	6	38.08	58.85	6 091 080	5.9	5351	36 623
Chr3	99 967 245	4	38.14	52.55	5 773 374	5.78	5066	36 000
Chr4	58 378 603	2	38.18	103.76	3 154 452	5.4	2824	22 667
Chr5	86 516 636	4	38.22	48.46	4 797 374	5.55	4367	31 021
Chr6	66 589 920	3	38.05	80.1	3 519 561	5.29	3122	24 855
Chr7	84 298 359	3	38.25	50.29	4 358 578	5.17	3862	32 023
Chr8	65 456 480	6	38.07	52.86	3 330 112	5.09	3058	23 921
Chr9	53 798 904	2	37.9	62.16	2 892 269	5.38	2667	20 190
Chr10	51 931 824	5	38.14	63.65	2 600 983	5.01	2450	21 024
Chr11	55 055 587	5	38.15	66.74	2 926 018	5.31	2601	20 696
Chr12	50 913 815	3	38.15	66.93	2 378 917	4.67	2153	25 008
Chr0	39 081 123	1015	49.27	74.75	5 859 891	14.99	6434	47 033
Total 12 Chr	873 906 699	48	38.14	62.09	47 394 434	5.42	42 481	329 979

Supplementary Tools S1: list of tools/softwares used.

### Avocado genome synteny

**Figure 2:**
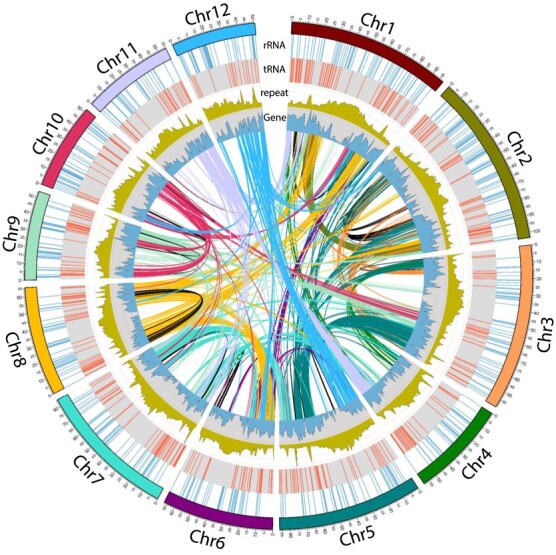
Circos plot indicating chromosomes on the outer track followed by band graphs of rRNA (blue) and tRNA (red) counts moving inward. Line plots for repeats (olive) and genes (blue) indicate the abundance of corresponding features in million bp bins. The innermost links indicate genomic block duplications (stemming from WGDs), with colours indicating the chromosome of origin except for self-synteny, which is represented in black. rRNAs are concentrated on chromosome arms near centromeres, whereas tRNAs are more abundant toward telomeric regions. Repeats are also highly distributed at centromeric regions of chromosomes. The regions with higher numbers of repeats contain fewer genes. Chr1,5,3 and 2 had largest synteny, whereas Chr2,0 and 8 had largest self-synteny.

Several duplications and inversion events were found using a self-self syntenic comparison. Synteny plots represent the physical co-localization of genes on chromosomes. These plots revealed the two whole genome duplication events (WGDs) that had previously been uncovered by Rendon-Anaya et al. and from other sequenced Lauraceae genomes. In these dotplots, diagonal matches represent the syntenic orthologs and paralogs when comparing two different species (avocado vs. *Litsea*; Supplementary Fig. S7). When comparing different assembly versions of the same species, the diagonal gaps indicate insertions/additions of sequence (Supplementary Fig. S5 and S6) and the inverted regions indicate inversions, as shown in the self-self assembly (Figure 2 and Supplementary Fig. S5) and our assembly versus the genome from Rendon-Anaya et al. [43] (Supplementary Fig. S6). The syntenic blocks from self-self synteny obtained from CoGe were re-plotted using links in the Circos plot (Figure 2), where various large syntenic sections were observed among the chromosomes.

A number of macro- and micro-syntenic regions can be noted in the comparison, e.g. Chr12 showed a large block with macrosynteny to Chr5 (Supplementary Fig. S8). Inversion events were recorded on Chr1, 2, 5, 8 and 12. A large part of Chr2 near the centromere was duplicated on the other arm, whereas a region near one of the telomeric ends was inverted on Chr1. Similarly, duplicated regions on Chr3 were observed on Chr5 and Chr9. Chr5 also had duplicated regions on Chr3 and Chr7 and inverted regions on Chr2, 3 and 12. Another major duplication was observed on Chr7, where almost all of one arm was found in duplicate form on Chr5. Chr8 had a duplicate region on Chr1. Chr11 had an inversion and duplication on the second arm of Chr1. Similarly, Chromosome 12 held an inversion and duplication on one of the arms of Chr5. Self synteny suggested a higher number of gene duplications on Chr3 and Chr5 with respect to other chromosomes. The genes with multiple copies were found to be involved in processes such as regulation of transcription, protein phosphorylation, protein ubiquitination, transmembrane transport, proteolysis, phosphorylation, translation, and carbohydrate metabolic process.

*L. cubeba* is a close phylogenetic relative of avocado. Conservation of synteny between *Litsea* and avocado was observed on various chromosomes, such as avocado Chr1, Chr2, Chr5, Chr9 and Chr11. Conserved but inverted synteny can be noted for Chr4, Chr6, Chr7 and Chr12 (Supplementary Fig. S7). Finally, it cannot be excluded that some of the inversions that we or others have noted in avocado and other Lauraceae could be due to incorrect contig orientation from map- or HiC-based scaffolding procedures.

### Repeat analysis

Searching against the eukaryote repeat database, Dfam_3.1 using RepeatMasker V. 4.1.0, 28 Mb (3.09%) of the assembled genome was masked (Supplementary Table S3). RepeatModeler V. 2.0.3, an *ab-initio* repeat masking tool, was used to mask other unknown repeats from the assembled genome. *Ab-Initio* predictions generally have difficulty assigning repeats into sub-categories, but with the current version of RepeatModeler [18], more categories have been assigned. *Ab-initio* prediction masked 551 Mb of the genome. In total, 571.8 Mb (62% of the assembled genome) was masked, out of which the 12 chromosomes included 542.6 Mb of repeat regions (Table 1 and Supplementary Table S3).

Helitrons were found in a higher ratio with respect to chromosome size for Chr12, 10, 7 and 1, whereas Chr1, 7, 2 and 5 had greater counts of these repeats (Supplementary Fig. S4). Chr12 had the highest ratio (22.24%), whereas Chr1 had the greatest total length (18.7 Mb, 19.7%) of long terminal repeat (LTR)/Caulimovirus repeats (Supplementary Fig. S4).

### Annotation

Annotation with BRAKER V. 2.1.6 [4] using available RNA-Seq data enabled prediction of 46 147 genes from which 11 788 were single-exonic. The annotation identified totals of 228 370 exons and 168 150 introns. The gene density was similar for all chromosomes and ranged from 4.7–5.9% with respect to chromosome sizes. The average coding region length associated to chromosomes 1–12 was similar (~1100 bp). The annotation was estimated to be 96.6% complete by BUSCO analysis with respect to the Embryophyta lineage. The gene length ranged from 39 to 83 886 and the N50 length was 1515 bp. The genes were mostly identified in regions with no repeats (Figure 2). Coding potential estimation categorized 42 796 genes as coding and 6119 as non-coding.

A blastx search using plant protein databases identified 39 207 transcript hits with an e-value cut-off of 1e-10. The largest number of blast hits (30250) were from *Cinnamomum micranthum* f. *kanehirae*. A very high number of transcripts were associated with pol (DNA polymerase) polyprotein, serine/threonine-protein kinase and phosphatase, and disease resistance protein.

Protein family classification was assigned to 33 663 transcripts, where 16 320 InterPro terms were associated. Protein kinase, zinc finger, serine–threonine kinase, RNA recognition motif and leucine-rich repeats were among the most prominent domains. Functions associated with a large number of gene sequences or having a high copy number are generally common between plant species as they are associated with core plant functions [31]. Genes related to enzymes oxidoreductases, transferases and hydrolases had a high copy number in avocado.

Thirteen genes were annotated as RNase H family proteins, which are essential for degrading RNAs that base-pair with DNA. Several RNase H associated genes were located on 9 chromosomes (Chr6, 9 and 12 contained 2 copies).

377 012 ORFs (Open Reading Frames) containing start and stop codons were identified in the pseudo-assembled genome using the EMBOSS getorf tool with a length cut-off of 300 bases. Of these, 329 979 ORFs belonged to the 12 chromosomes. From these, the longest ORF was on Chr7 and measured 9914 bases, whereas 95 903 ORFs had lengths of more than 500 bases.

Barrnap identified 4146 ribosomal RNA (rRNA) genes in the genome. A ribosomal gene cluster was located on Chr2 (Figure 2). Interestingly 5 s rRNA genes were abundant in all chromosomes, unlike some other plants where they are located only on certain chromosomes [48]. Chr12 and Chr0 contained a greater number of 18 s, 28 s and 8 s rRNA gene sites than 5 s rRNA gene sites.

1786 tRNA sites (tDNA) were identified, of which 497 were located on 12 chromosomes (Figure 2).

Subcellular localization analysis predicted 3177 signal peptides, 1230 chloroplast transit peptides (cTP), 108 thylakoid luminal transit peptides (luTP) and 1058 mitochondrial transit peptides (mTP) from the proteome, based on plant models.

Signal peptides regulate the entry of proteins to the secondary pathway and are found in secreted and transmembrane (TM) proteins, as well as in proteins inside organelles in eukaryotic cells [1, 40]. Signal peptide cleavage site detection resulted in the identification of 2587 signal peptides using the eukaryotic model.

**Figure 3:**
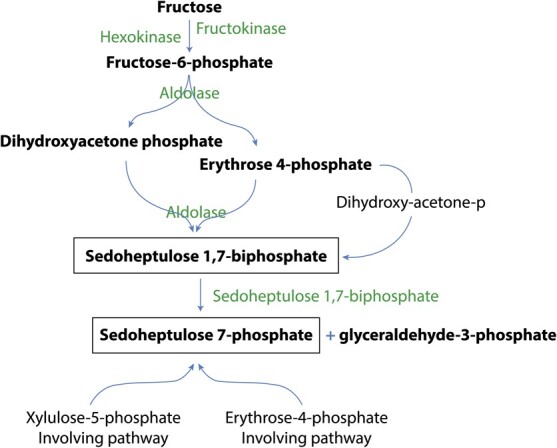
Sedoheptulose 7-phosphate pathway, the most likely pathway for heptose sugar biosynthesis in avocado (pathway adapted from Raines et al. [42]). The reaction products are represented in bold whereas the final product (heptose) is represented inside the box.

### Sugars in avocado

Primary metabolism in avocado involves 7-carbon sugars. Heptose sugars are common to gram-negative bacteria but are rare in plants. In addition to *Medicago sativa* (alfalfa), *Kalanchoe pinnata*, and plants from the genus *Primula* [15] avocado also produces heptose. The genes regulating heptose sugar biosynthesis pathways (Figure 3) were investigated in the assembled avocado genome. An analysis of Hierarchical Orthologous Groups (HOGs) among avocado, *Cinnamomum micranthum* and tomato (*Solanum lycopersicum*) demonstrated evolutionary expansion and contraction of gene families involved in this pathway. Avocado gained genes for the sedoheptulose-1,7-biphosphatase (3), hexokinase (3), fructose biphosphate (5) and fructokinase (3) families compared to the *Cinnamomum* and tomato, with duplications relative to the common ancestor of avocado and *Cinnamomum* also evident (Supplementary Figs. S11-S14). In contrast to *Cinnamomum*, no gene loss in these families was identified for avocado.

**Figure 4:**
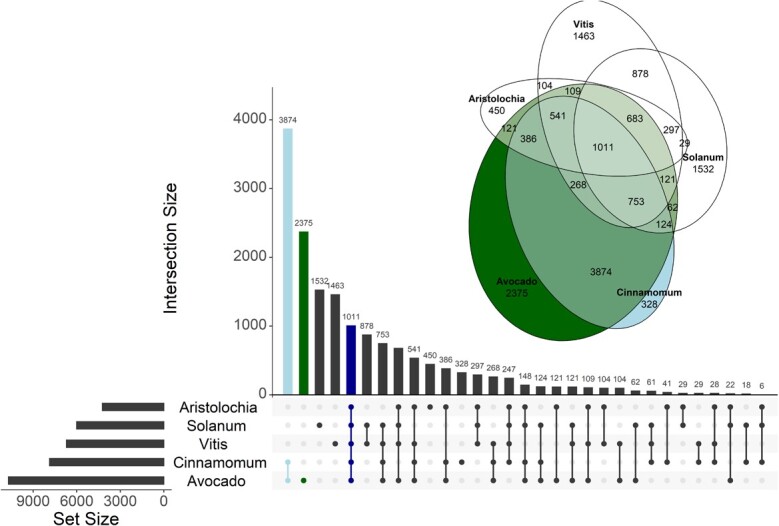
Gene clustering between 5 plant species (Persea amaricana, *Solanum lycopersicum*, *Vitis vinifera*, Aristolochia fimbriata and Cinnamomum micranthum). The bar graph represents the count of sub-clusters for the combinations indicated underneath the graph. The bars corresponding to the avocado and Cinnamomum cluster, cluster specific to avocado, and the cluster containing common sub-clusters to all five species are highlighted in light blue, green and blue respectively. The Euler diagram indicates weighted sub-cluster count for five species, where avocado is represented in green and Cinnamomum, the closely related species, is represented in light blue.

The expression of genes involved in the heptose sugar biosynthesis pathways was investigated using RNA-Seq data derived from fruit tissues (Supplementary Table S5). This analysis indicated that most genes identified as members of these pathways were indeed expressed in fruit. Additional analysis of different fruit development stages may also reveal expression for those genes which were not expressed in these samples.

### Fruit ripening in avocado

Avocado fruit ripening is associated with softening due to the action of cellulases, while the softening of most other fruit is usually associated with the action of polygalacturonases on pectic cell wall polysaccharides [52]. Investigation of genes regulating cellulase degradation in the assembled genome revealed that endoglucanase and endo-polygalacturonase were more abundant (two or more times as many copies) compared to the number found in Cinnamomum.

### Potassium channels and transporter in avocado

Avocado is rich in potassium [34], similar to *Cinnamomum*. Ion transporters were comparable in number to those reported in *Cinnamomum*. The similar copy number for most of the genes involved in potassium biosynthesis and transport may be associated with the high potassium content in both plants.

### Orthologous genes in avocado and other species

Proteins from five plant species (avocado (Lauraceae), *Solanum* (shares cellulase based ripening), *Vitis* (Rosids), *Aristolochia* (basal angiosperm) and *Cinnamomum* (Lauraceae)), were clustered to identify unique and shared genes (Figure 4, Supplementary Fig. S9). 8152 genes in 2375 clusters were found unique to avocado. These orthogroups were involved in pollen recognition, DNA recombination and the terpenoid biosynthetic process and were enriched for transferase, hydrolase, ion binding and oxidoreductase activities.

14, 392 orthogroups from avocado were common to all the species. 5099, 193, 181 and 150 genes were clustered uniquely with *Cinnamomum*, *Aristolochia*, *Vitis* and *Solanum* respectively, consistent with the closer evolutionary relationship between *Cinnamomum* and avocado. The largest sub-clusters unique to avocado and *Cinnamomum* were involved in regulation of transcription and defence response. Avocado, *Cinnamomum* and *Aristolochia* are all magnoliids. This cluster contained genes involved in RNA modification, mannose binding and protein dimerization activity. Avocado shares cellulase-based fruit ripening with *Solanum*. The cluster between avocado and *Solanum* was found to be involved in DNA integration and regulation of growth.

Based on the HOG analysis of avocado, *Cinnamomum* and tomato, 22 355 genes were gained compared to the reconstructed ancestral genome of *Cinnamomum* and avocado, with 17 471 genes retained, 6321 duplicated and 1651 lost (Supplementary Fig. S10).

## Discussion

Here, we have reported the first haploid-resolved assembly of avocado, from the magnoliid family Lauraceae. Leveraging high-quality, long HiFi reads, the sequences of the two haplotypes were reconstructed. The sequence length difference between the haplotypes was low for most chromosomes (Supplementary Fig. S4). The primary genome assembly produced using the Hifiasm tool had a very high N50 value relative to the previous assembly by Rendon-Anaya et al. [43] (26 Mb versus 296 kb).

Our initial assembly also contained organellar contigs. To remove them, organellar genomes (mitochondrial and plastid) were assembled (Fig. 1) and screened from the nuclear genome assembly. In avocado, the plastid genome was of intermediate size (153 kb) with respect to the known range of plastid genomes, i.e. 100–220 kb [45]. Mitochondrial genomes in plants can vary greatly in size (100–10 000 kb), with the size in avocado being small compared to those of most flowering plants [17]. Song et al. [50] had previously assembled and reported the chloroplast genome of avocado; our genome assembled in this work is 9 bp longer, and the improved genome allowed more genes to be annotated than had been previously reported.

The Hifiasm contigs, when assembled into pseudochromosomes, formed 12 chromosomes and an unordered Chr0. This assembly is larger with respect to the earlier estimated Hass genome size, but 95.0% of our predicted avocado genome size (i.e. 920 Mb). The very few (48) contigs forming 12 pseudochromosomes and the closeness of the anchored genome length (873.9 Mb) to expected genome length (920 Mb) indicates that most of the assembled length was covered by a few long contigs. As the total contig length was close to the expected genome size the assembly can be considered to cover most of the genome.

Repeated sequences are a major part of eukaryotic genomes [3], and are responsible for some genomic novelties and evolution by epigenetic regulation [38]. Similar in proportion to many other plant species [32], 62% of the avocado genome comprises repeats. Helitrons are known for their ability to transfer horizontally between distantly related species and capture gene fragments during transposition, by a mechanism similar to rolling-circle replication via a single-stranded DNA intermediate [23]. Thus, a high number of Helitrons on avocado chromosomes may suggest an elevated role in the plasticity of this species. Many genes on Chr12 or Chr1 could be enriched for defense responses as they were rich in long terminal repeats (LTRs). LTR elements are widely distributed ancient genome elements but are especially abundant in plants [27]. They are known to play an important roles in genome size changes [27] and can impart immunity by creating functional disease-resistance genes from the process of retroduplication [19]. Chen et al. [13] reported LTR transposons to occupy 47.6% of the *Litsea cubeba* genome, a closely related Lauraceae species, whereas, in avocado, only 16.5% of the genome was occupied by LTR repeats.

The fewer gaps observed in syntenic dotplot analysis, when compared with the Rendon-Anaya et al. [43] genome assembly, strongly suggests capture of a greater proportion of the avocado genome sequence. Many paralogously duplicated blocks were noted, supporting Rendon-Anaya et al. [43] determination that the avocado lineage underwent two whole-genome duplication/polyploidization events. Genes with multiple copies were found to be involved in processes such as regulation of transcription, protein phosphorylation, protein ubiquitination, transmembrane transport, proteolysis, phosphorylation, translation, and carbohydrate metabolic process. Interspecific synteny with *Litsea* marked large conserved chromosomal sections between the genomes, indicating strong structural conservation in the Lauraceae family. The greater contiguity of our avocado genome assembly compared to that previously published might contribute to enhanced studies of magnoliid and basal angiosperm evolution.

The ratio of the number of genes present on each chromosome to the size of the chromosome was approximately the same for all chromosomes. Genes identified were mostly placed in regions lacking repeats (Figure 2).

Many genes were annotated with disease resistance function, which could be further investigated for enhancing avocado productivity. The cellular component analysis identified various genes associated with organelle and intracellular organelle trafficking between nucleus and organelles. RNAse H, a non-sequence specific endonuclease cleaving RNA of DNA/RNA hybrids, is found in most organisms [5]. The abundance of these domains in avocado points to a major role in viral defence in plants, and as a gene modifier and driver of evolution and genome diversity [37] in avocado.

Primary metabolism in avocado is unique, involving 7-carbon sugars and sugar alcohols [15]. Additionally, avocado is rich in heptose. The presence of a sedoheptulose 1,7-bisphosphatase suggests the predominant pathway of heptose biosynthesis in avocado is via sedoheptuloase 1,7 bisphosphate rather than alternative routes that do not involve this intermediate. The high copy number of heptose sugar synthesis regulating genes in avocado could help explain the abundance of heptose in avocado and its absence in *Cinnamomum*. The HOGs analysed among avocado, *Cinnamomum* and tomato indicated the evolutionary retention, duplication, and in particular, gain of genes related to the 7-carbon sugar pathway. Avocado also has a fruit ripening mechanism involving cellulase. Endo-polygalacturonase acts as a glycosidase and hydrolase, and is involved in maceration and soft-rotting of plant tissue [29]. The abundance of endoglucanase genes observed supports avocado using cellulase for fruit ripening.

The availability of a largely complete genome allows for the investigation of many other key pathways in avocado. Potassium content for some of the studied Lauraceae species is higher than in banana [8, 22]. Avocado when compared to *Cinnamomum* had a similar copy number of potassium metabolism-related genes, possibly reflecting similar potassium content. Investigations of the genes responsible for avocado’s unique lipid composition are also now feasible via genome mining in combination with gene expression analysis across genotypes and developmental stages. As a starting point to further exploration, we identified genes likely involved in the lipid metabolism pathway (Table S4).

The proteomes from five angiosperm genomes were clustered to identify unique and shared genes with respect to avocado. Many unique genes specific to avocado and Lauraceae were identified. Mannose binding-related orthogroups were enriched specifically in avocado and tomato, and contained cellulase genes that might reflect the common ripening processes in these species.

This high-quality avocado genome provides a key resource to support avocado genetic improvement. The availability of other high quality genomes from the mangoliids for comparative analysis in future will complement and improve understanding of early angiosperm evolution.

## Material and methods

### Sample collection

A mixture of young and mature leaves were collected from a clone of the mother Hass avocado tree, aged 30, planted at Maroochy Research Facility, Nambour, Queensland. The collected leaves were snap-frozen in liquid nitrogen, followed by transport on dry ice and stored at −80°C until further processing. An aliquot of frozen leaves was finely ground in liquid nitrogen using a mortar and pestle and stored at −80°C before being further used for nucleic acid extraction.

### Genomic DNA extraction and sequencing

Nucleic acid was extracted from the ground leaf tissues using a modified CTAB (cetyl-trimethyl ammonium bromide) based method [39]. The genome was sequenced using PacBio HiFi Sequencing at the Institute for Molecular Bioscience, The University of Queensland, Australia (Supplementary Table S1, Fig. S1). The DNA was fragmented, aiming at a peak fragment length of 17 kb using a Megaruptor® (two-step shearing with a 25 kb setting) and sequenced on three SMRT cells. The genome was also sequenced using Illumina Nextra-seq sequencing on an S4 lane at Ramaciotti Centre for Genomics, NSW, Australia at an expected coverage of 100x and 150 bp length.

### RNA extraction and sequencing

Various tissue types were collected from a mature avocado tree cultivar Hass located at the Department of Agriculture and Fisheries (DAF), Maroochy Research Facility (Nambour, Australia). Tissue types included leaves, roots, fruit, inflorescences and stems. Samples were also collected from tissue culture propagated and glasshouse raised plants and included intact seedling, roots, callus, leaves and stems [25, 39]. Upon harvesting, samples were immediate snap-frozen in liquid nitrogen prior to transportation.

RNA was extracted from the individual tissues using a modified CTAB based method [39]. The RNA extracted from all the tissues were pooled in equal amounts and sequenced using Illumina NovaSeq paired-end short read sequencing at an expected 100x coverage [39].

### Draft genome assembly

The sequenced PacBio HiFi reads were quality assured using SMRT Link v9.0 by verifying adaptor or short insert contaminations followed by consensus read calling. The consensus reads were assembled using Hifiasm [14] for High-Fidelity (HiFi) long reads. Next, assemblies were assessed using the Quality Assessment Tool (QUAST) v5.0.2 based on a contig size >500 bp [21, 36] and seqstats v1.0.0 (https://github.com/clwgg/seqstats), and completeness was evaluated using BUSCO v5.2.2 with the embryophyta_odb10 database [47].

Additionally, Illumina reads were used to assemble the plastid and mitochondrial genomes of avocado using the GetOrganelle tool [28].

### Assembly of pseudomolecules

The assembled HiFi reads were screened for organellar genomes via Blast [11] using the assembled plastome and mitochondrial genomes as well as a previously published chloroplast genome [50]. Sequences, where more than 80% of the contigs were covered with an expected value 1e-5 were removed. The remaining contigs were scaffolded using SynMap [24] in CoGe [33] to build into a chromosome level assembly based on CDS from the marker-based anchored genome from Rendon-Anaya et al. [43]. To assess the quality and accuracy of the assembled genome, Inspector v.1.0.2 [12] was used. The PacBio HiFi reads were mapped using Inspector tool to the assembled genome. The output included a QV score (Quality Value), indicating the accuracy of the assembled genome.

### Annotation

Knowledge-based repetitive elements were discovered and masked using RepeatMasker V. 4.1.0 [49]. The masked sequences were further analysed using RepeatModeler V. 2.0.3 [18] for *ab-initio* repeat identification.

To identify structural features, HISAT2 v.2.1 [30, 41] was used for mapping avocado RNA-Seq data from SRR1463207 (mesocarp Pool: Stages I to V), SRR9595469 (Day 1: Fruit with oviposited eggs), SRR8926016 (RNA-Seq from different parts), SRR8991204 (1 month old: juvenile plant), SRR12391891 (Infected stem: 14 days post infection), SRR12391884 (un-infected stem), SRR9595486 (avocado fruit without any obvious damage), SRR12566298 (IBA de-etiolated shoot and apical meristem), SRR8926017 (RNA-Seq of different parts), SRR5738276 (fruit pulp), SRR1463349 (mesocarp Stage IIIb), SRR9026409 (mesocarp: 145 days post full bloom), SRR9026405(Seed: 110 days post full bloom), SRR2000041 (Mixed library: organs and ripening stages), and SRR2000042 (Mixed library: organs and ripening stages) obtained from Sequence Read Archive (SRA) database and an in-house sequenced RNA-Seq reads to the soft-repeat masked pseudo-assembled genome. The SRA samples were selected from multiple sources, considering fewer representative samples (few samples selected from various projects).

The automated genome annotation pipeline BRAKER V. 2.1.6 [4] was used to infer structural features. GenSAS [26] was used for ORF detection, and signal and target peptide identification. The agat [16] tool was used to obtain CDS and protein sequences from the GFF (generic feature format) file. CDS regions were functionally annotated using OmicsBox v2.0 (BioBam Bioinformatics, Valencia, Spain). This used a BLASTX homology search with a maximum of 10 hits against the NCBI non-redundant protein database, subset viridiplantae at an e-value of 1e-10. The GO terms were assigned to the transcripts. InterProScan was run using various domain databases, to classify proteins into families and in predicting domains and other sites.

Organellar genomes were annotated using GeSeq [51] and plotted using OGDRAW [20].

### Genome-wide gene clustering

Five plants were selected for orthogroup clustering analysis including *Persea amaricana* (Lauraceae), *S. lycopersicum* (dicot, Solanaceae, shares cellulase based ripening), *V. vinifera* (dicot, Rosids), *Aristolochia fimbriata* (dicot, basal angiosperms diverged close to the divergence of monocots from dicots), *Cinnamomum micranthum* (Lauraceae). Protein sequences were clustered at e-value 1e-5 using OrthoVenn2 [54]. HOG analysis was carried out using OMA Standalone and Pyham for visualisation [2].

## Acknowledgements

The authors wish to acknowledge the help from the Maroochy Research Facility (47 Mayers Rd, Nambour QLD 4560) for providing avocado samples for use in this work. We acknowledge Victor A. Albert for his guidance, supervision and editing this manuscript. The authors acknowledge the University of Queensland Research Computing Centre (UQ-RCC) for providing all the computing resources.

This research received funding from Hort Innovation, with The University of Queensland and the Australian Government as part of the National Tree Genomics Program, AS17000 Genomics Toolbox. ON was supported by a graduate scholarship from The University of Queensland.

## Contributions

ON designed and conducted the experiments, performed analysis, curated data, and wrote the manuscript. NM, RJH, SJF, AH, AF, AKM and LMS advised on design, analysis and data interpretation. All authors read, edited and approved the final manuscript.

## Data availability statement

All data generated or analysed during this study are included in this published article. All data and materials used and described in this study are made available for non-commercial research purposes. The data that support the findings of this study are openly available in Sequence Read Archive (SRA) under the BioProject number PRJNA694184 and PRJNA818813.

## Conflict of interests

The authors declare that they have no competing interests.

## Supplementary data

Supplementary data is available at *Horticulture Research* online.

## Supplementary Material

Web_Material_uhac157
