# Supplementary material for: A haplotype resolved chromosomal level avocado genome allows analysis of novel avocado genes
Source: Hortic Res. 2022 Aug 1;9:uhac157. doi: 10.1093/hr/uhac157 (PMC9531333; doi:10.1093/hr/uhac157)
Supplement: Web_Material_uhac157 [file web_material_uhac157.docx]

# Supplementary Tools S1: list of tools/software used

| Tool | Used for | Reference |
| --- | --- | --- |
| HiFiasm | Genome Assembly | (Cheng;2021;1) |
| GetOrganelle | Organellar Genome assembly | (Jin;2020;2) |
| SynMap (CoGe) | Synteny analysis and pseudochromosome assembly | (Haug-Baltzell;2017;3) (Lyons and Freeling;2008;4) |
| QUAST | Quality assessment | (Gurevich;2013;5, Mikheenko;2018;6) |
| seqstats | Fasta sequence statistics | (https://github.com/clwgg/seqstats) |
| Busco | Genome assembly QC | (Seppey;2019;7) |
| Blast | Annotation | (Chen;2015;8) |
| RepeatMasker | Repeat discovery | (Smit;2019;9) |
| RepeatModeler | Repeat discovery | (Flynn;2020;10) |
| HISAT2 | Mapping | (Kim;2019;11, Pertea;2016;12) |
| BRAKER | Annotation | (Brůna;2021;13, Hoff;2016;14, Hoff;2019;15, Li;2009;16, Barnett;2011;17, Lomsadze;2014;18, Buchfink;2015;19, Stanke;2008;20, Stanke;2006;21) |
| GenSAS | Annotation | (Humann;2019;22) |
| Agat | Fasta from gff | (Dainat;2021;23) |
| GeSeq | Organelle annotation | (Tillich;2017;24) |
| OGDRAW | Organelle plotting | (Greiner;2019;25) |
| OrthoVenn2 | Clustering | (Xu;2019;26) |
| Barrnap | Ribosomal RNA identification | (Seemann;2018;27) |

# References

1. Cheng HY, Concepcion GT, Feng XW, Zhang HW, Li H. Haplotype-resolved de novo assembly using phased assembly graphs with hifiasm. Nat Methods. 2021;18(2):170-+.

2. Jin JJ, Yu WB, Yang JB, Song Y, dePamphilis CW, Yi TS, et al. GetOrganelle: a fast and versatile toolkit for accurate de novo assembly of organelle genomes. Genome Biol. 2020;21(1).

3. Haug-Baltzell A, Stephens SA, Davey S, Scheidegger CE, Lyons E. SynMap2 and SynMap3D: web-based whole-genome synteny browsers. Bioinformatics. 2017;33(14):2197-8.

4. Lyons E, Freeling M. How to usefully compare homologous plant genes and chromosomes as DNA sequences. Plant J. 2008;53(4):661-73.

5. Gurevich A, Saveliev V, Vyahhi N, Tesler G. QUAST: quality assessment tool for genome assemblies. Bioinformatics. 2013;29(8):1072-5.

6. Mikheenko A, Prjibelski A, Saveliev V, Antipov D, Gurevich A. Versatile genome assembly evaluation with QUAST-LG. Bioinformatics. 2018;34(13):142-50.

7. Seppey M, Manni M, Zdobnov EM. BUSCO: Assessing Genome Assembly and Annotation Completeness. Gene Prediction: Methods and Protocols. 2019;1962:227-45.

8. Chen Y, Ye WC, Zhang YD, Xu YS. High speed BLASTN: an accelerated MegaBLAST search tool. Nucleic Acids Res. 2015;43(16):7762-8.

9. Smit A, Hubley R, Green P. 2013–2015. RepeatMasker Open-4.0. 2019.

10. Flynn JM, Hubley R, Goubert C, Rosen J, Clark AG, Feschotte C, et al. RepeatModeler2 for automated genomic discovery of transposable element families. Proc Natl Acad Sci U S A. 2020;117(17):9451-7.

11. Kim D, Paggi JM, Park C, Bennett C, Salzberg SL. Graph-based genome alignment and genotyping with HISAT2 and HISAT-genotype. Nat Biotechnol. 2019;37(8):907-+.

12. Pertea M, Kim D, Pertea GM, Leek JT, Salzberg SL. Transcript-level expression analysis of RNA-seq experiments with HISAT, StringTie and Ballgown. Nat Protoc. 2016;11(9):1650-67.

13. Brůna T, Hoff KJ, Lomsadze A, Stanke M, Borodovsky M. BRAKER2: Automatic eukaryotic genome annotation with GeneMark-EP+ and AUGUSTUS supported by a protein database. NAR genomics and bioinformatics. 2021;3(1):lqaa108.

14. Hoff KJ, Lange S, Lomsadze A, Borodovsky M, Stanke M. BRAKER1: Unsupervised RNA-Seq-Based Genome Annotation with GeneMark-ET and AUGUSTUS. Bioinformatics. 2016;32(5):767-9.

15. Hoff KJ, Lomsadze A, Borodovsky M, Stanke M. Whole-Genome Annotation with BRAKER. Gene Prediction: Methods and Protocols. 2019;1962:65-95.

16. Li H, Handsaker B, Wysoker A, Fennell T, Ruan J, Homer N, et al. The Sequence Alignment/Map format and SAMtools. Bioinformatics. 2009;25(16):2078-9.

17. Barnett DW, Garrison EK, Quinlan AR, Stromberg MP, Marth GT. BamTools: a C++ API and toolkit for analyzing and managing BAM files. Bioinformatics. 2011;27(12):1691-2.

18. Lomsadze A, Burns PD, Borodovsky M. Integration of mapped RNA-Seq reads into automatic training of eukaryotic gene finding algorithm. Nucleic Acids Res. 2014;42(15).

19. Buchfink B, Xie C, Huson DH. Fast and sensitive protein alignment using DIAMOND. Nat Methods. 2015;12(1):59-60.

20. Stanke M, Diekhans M, Baertsch R, Haussler D. Using native and syntenically mapped cDNA alignments to improve de novo gene finding. Bioinformatics. 2008;24(5):637-44.

21. Stanke M, Schoffmann O, Morgenstern B, Waack S. Gene prediction in eukaryotes with a generalized hidden Markov model that uses hints from external sources. BMC Bioinformatics. 2006;7.

22. Humann JL, Lee T, Ficklin S, Main D. Structural and Functional Annotation of Eukaryotic Genomes with GenSAS. Gene Prediction: Methods and Protocols. 2019;1962:29-51.

23. Dainat J. AGAT: Another Gff Analysis Toolkit to handle annotations in any GTF/GFF format (Version v0.8.0). 2021.

24. Tillich M, Lehwark P, Pellizzer T, Ulbricht-Jones ES, Fischer A, Bock R, et al. GeSeq - versatile and accurate annotation of organelle genomes. Nucleic Acids Res. 2017;45(W1):W6-W11.

25. Greiner S, Lehwark P, Bock R. OrganellarGenomeDRAW (OGDRAW) version 1.3.1: expanded toolkit for the graphical visualization of organellar genomes. Nucleic Acids Res. 2019;47(W1):W59-W64.

26. Xu L, Dong ZB, Fang L, Luo YJ, Wei ZY, Guo HL, et al. OrthoVenn2: a web server for whole-genome comparison and annotation of orthologous clusters across multiple species. Nucleic Acids Res. 2019;47(W1):W52-W8.

27. Seemann T. barrnap 0.9: BAsic Rapid Ribosomal RNA Predictor. Google Scholar. 2018.

28. Rendon-Anaya M, Ibarra-Laclette E, Mendez-Bravo A, Lan TY, Zheng CF, Carretero-Paulet L, et al. The avocado genome informs deep angiosperm phylogeny, highlights introgressive hybridization, and reveals pathogen-influenced gene space adaptation. Proc Natl Acad Sci U S A. 2019;116(34):17081-9.

# Supplementary S1

Table S1: Avocado DNA quality and quantity statistics for the samples sent for HiFi and Illumina sequencing.

| Sample | A260/A280 | A260/A230 | Conc ng/μl  (based on gel intensity) | Total DNA Quantity in μg |
| --- | --- | --- | --- | --- |
| HiFi | 1.97 | 1.82 | 301 | 60.20 |
| Illumina | 1.88 | 1.21 | 28 | 2.8 |


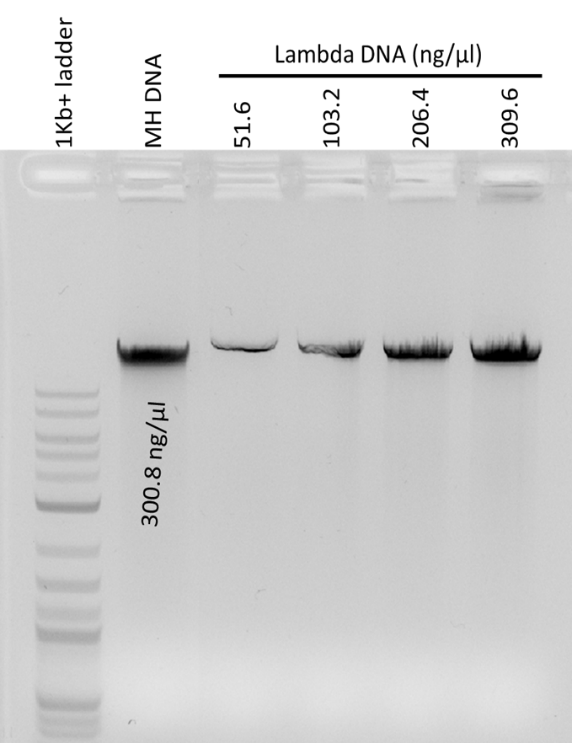

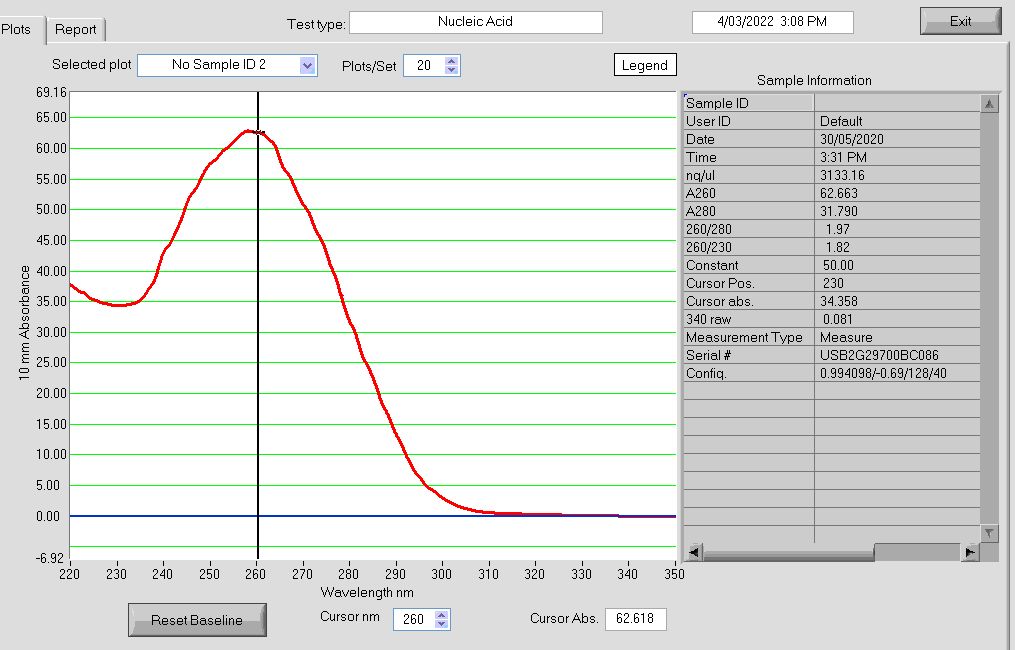


Figure S1: Agarose gel image and Nanodrop curve for DNA used for HiFi and Illumina Sequencing. A) Agarose gel image indicates high quantity of DNA as compared to lambda DNA standard B) The Nanodrop curve indicating high quality of DNA.

Table S2: Sequenced HiFi and Illumina read statistics.

| Sample | ccs size (GB) | Read Count | Total bases | N50 |
| --- | --- | --- | --- | --- |
| HiFi 1_A01 | 92 | 1,432,886 | 24,996,654,943 | 17,697 |
| HiFi 3_C01 | 31 | 491,923 | 8,477,600,085 | 17,481 |
| HiFi 4_D01 | 39 | 624,222 | 10,594,932,247 | 17,195 |
| Short Read _F | - | 406,108,367 | 60,944,893,164 | 151 |
| Short Read _R | - | 406,108,367 | 60,932,167,674 | 151 |


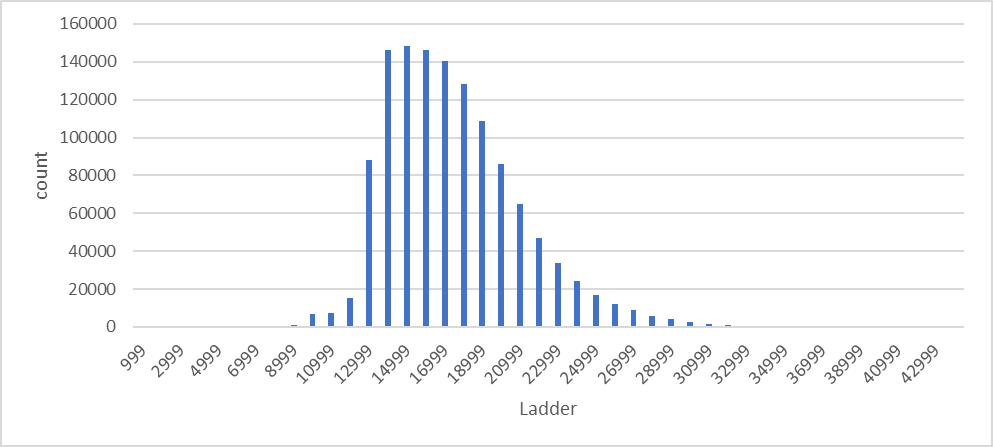


Figure S2: Length distribution of sequenced Hifi reads. The peak indicates higher number of reads sequenced at 15kb length, as expected.


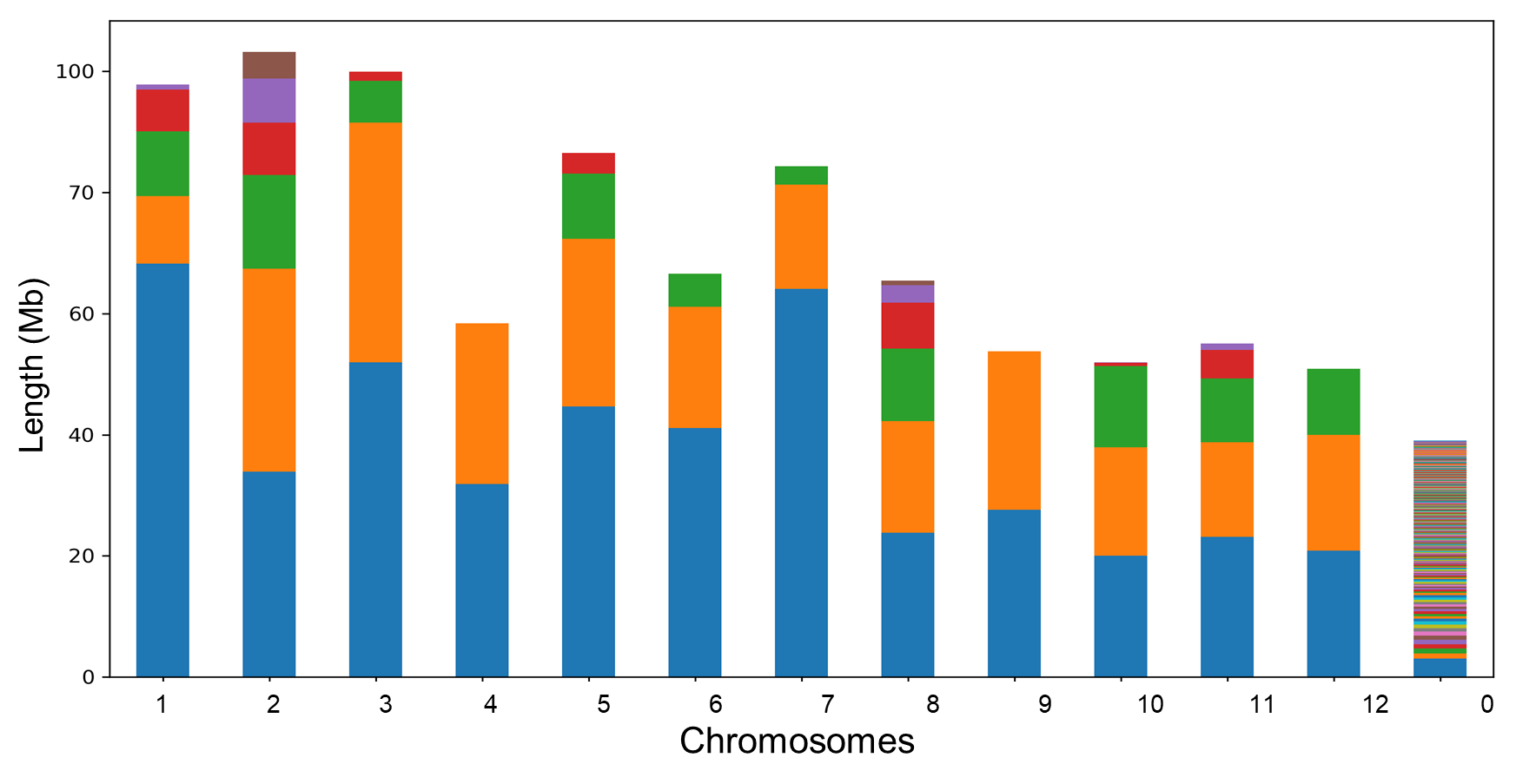


Figure S3: Length Distribution of pseudochromosomes from primary assembly. The stacked column graph indicate number of contigs forming chromosome stacked in descending order of size.


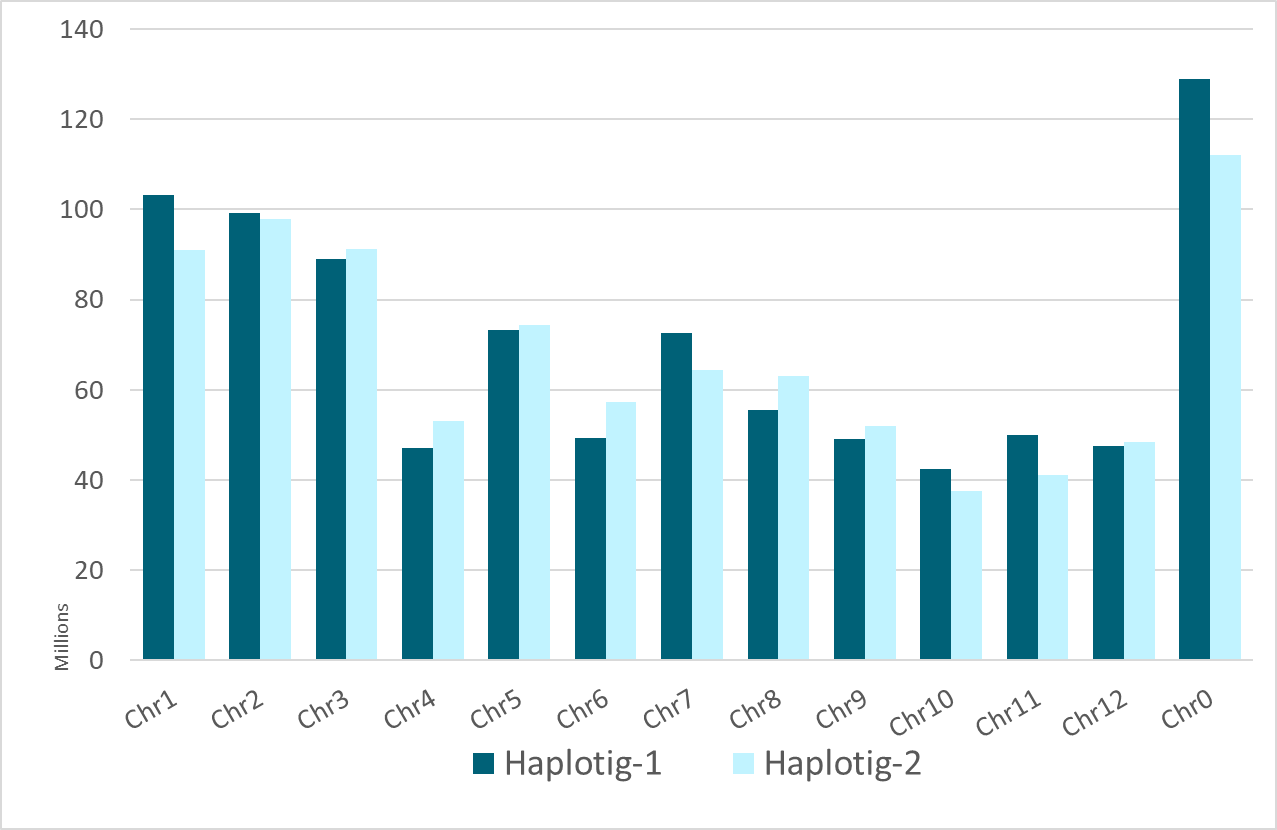


Figure S4: Length Distribution of haplotype resolved pseudochromosomes. The lengths of the two haplotigs are similar for most chromosomes.

Table S3: Repeat distribution. The values corresponding to RepeatMasker indicates repeats identified by knowledge-based approach whereas the values corresponding to RepeatModeler represent ab-initio based repeat discovery.

|  | RepeatMasker | | | RepeatModeler | | | Total | | |
| --- | --- | --- | --- | --- | --- | --- | --- | --- | --- |
| Element type | # elements | Length  Occupied | % of  Genome | # elements | Length  Occupied | % of  Genome | # elements | Length  Occupied | % of  Genome |
| Retroelements | - | - | - | 171485 | 202370167 | 22.86 | 171485 | 202370167 | 22.86 |
| SINEs | 121 | 11628 | 0 | 0 | 0 | 0 | 121 | 11628 | 0 |
| LINEs | 6239 | 958297 | 0.1 | 66334 | 55164863 | 6.23 | 72573 | 56123160 | 6.33 |
| LTR elements | 11120 | 3278759 | 0.36 | 105151 | 147205304 | 16.63 | 116271 | 150484063 | 16.99 |
| DNA transposons | - | - | - | 29293 | 20478542 | 2.31 | 29293 | 20478542 | 2.31 |
| DNA elements | 746 | 283814 | 0.03 | - | - | - | 746 | 283814 | 0.03 |
| Rolling-circles | - | - | - | 5147 | 9121839 | 1.03 | 5147 | 9121839 | 1.03 |
| Unclassified | 30 | 4298 | 0 | 905652 | 317237888 | 35.83 | 905682 | 317242186 | 35.83 |
| Total interspersed repeats | - | 4536796 | 0.5 | - | 540086597 | 61 | - | 544623393 | 61.5 |
| Small RNA | 5058 | 3221196 | 0.35 | 1859 | 1129305 | 0.13 | 6917 | 4350501 | 0.48 |
| Satellites | 69 | 6397 | 0 | 899 | 456920 | 0.05 | 968 | 463317 | 0.05 |
| Simple repeats | 414206 | 16165230 | 1.77 | 2479 | 146846 | 0.02 | 416685 | 16312076 | 1.79 |
| Low complexity | 77770 | 4236800 | 0.46 | 713 | 59560 | 0.01 | 78483 | 4296360 | 0.47 |
| **Total Repeats identified** |  | **28168009** | **3.09** |  | **551001067** | **62.23** |  | **571831504** | **62.63** |


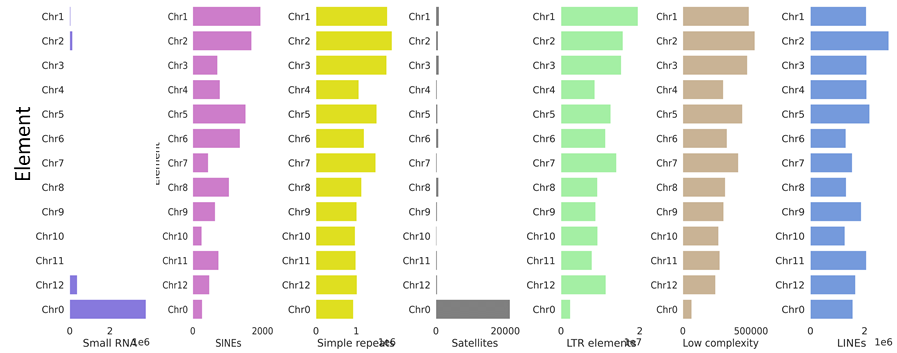

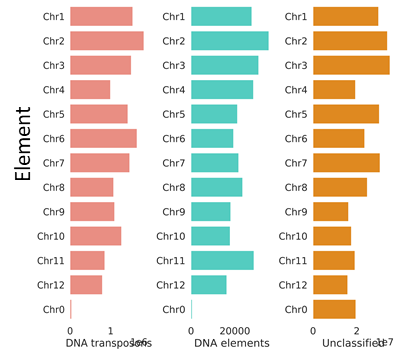

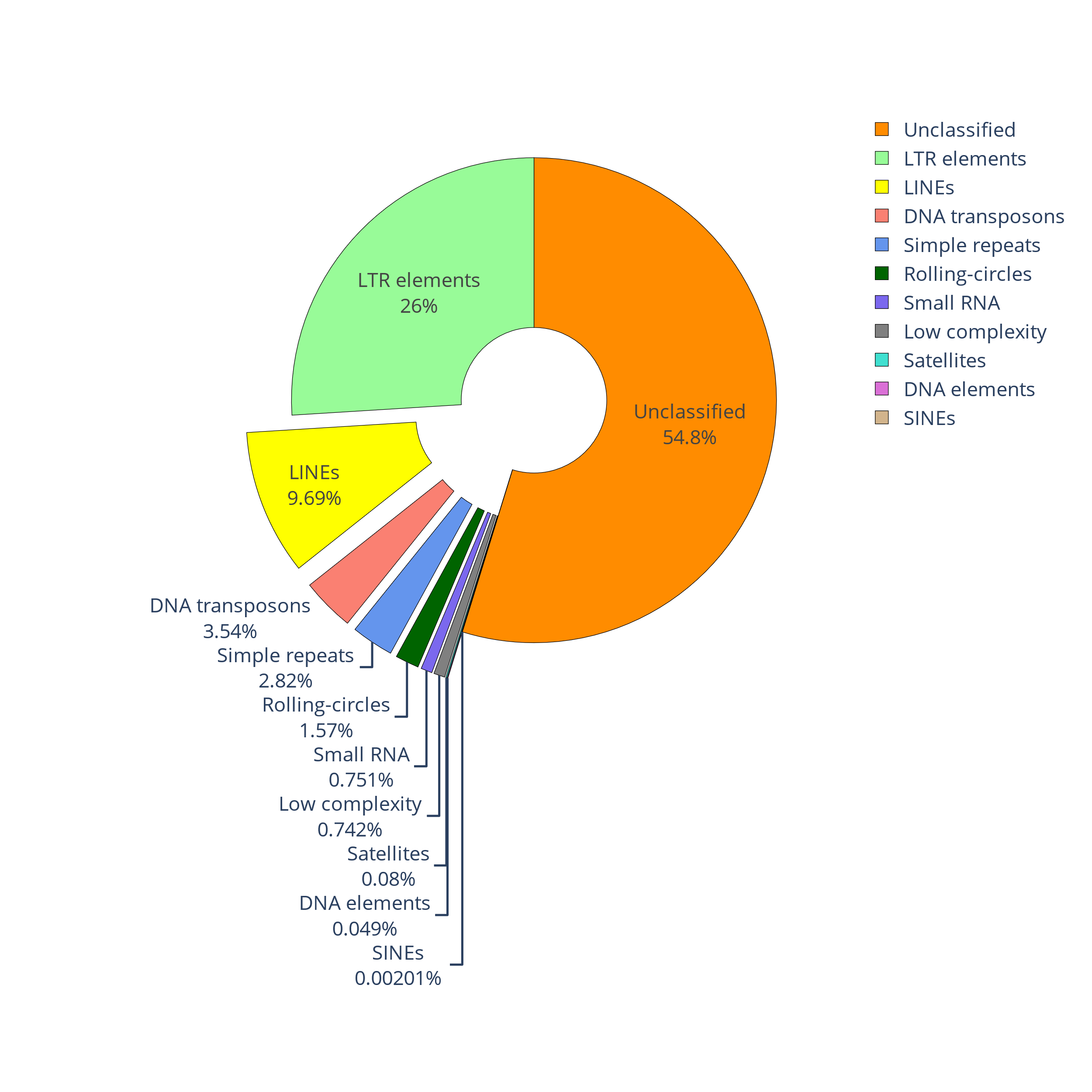


Figure S4: Repeat Distribution. The bar graphs indicate chromosome wise distribution of repeats. The pie graph indicates percentage distribution of repeat categories.


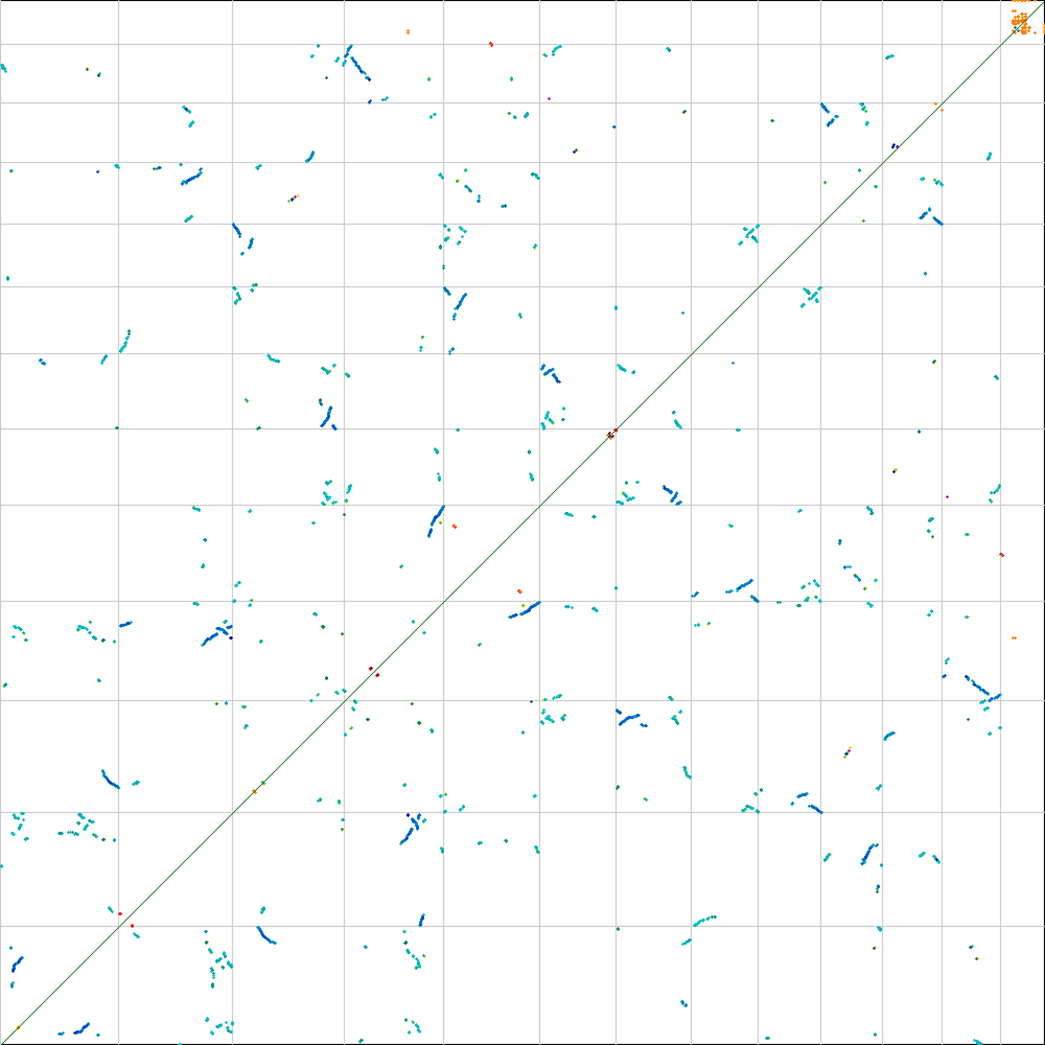


Figure S5: Self-Self synteny of pseudo assembled genome. Many inversions and duplications are plotted on graph. The colours originated by synonymous substitution rate calculation by CodeML.


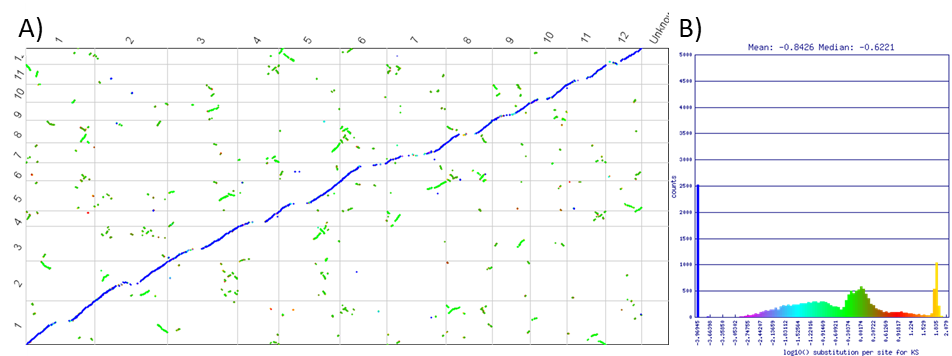


Figure S6: A) Synteny map of HIFI assembly Vs anchored pseudo-scaffolded 12 chromosomes from Rendon-Anaya (2019). As the two assemblies are different versions of same species, the diagonal gaps indicate insertions/additions of sequence and the inverted regions indicate inversions B) Synonymous substitution rate (KS) plot indicates avocado underwent two whole genome duplication events. The colours correspond to the colours in the synteny dot-plot, which originated by synonymous substitution rate calculation by CodeML. The blue peak corresponds to the perfect orthologs, whereas the red and green ones are paralogous blocks from a whole-genome duplication event; both at higher Ks corresponding to their age.


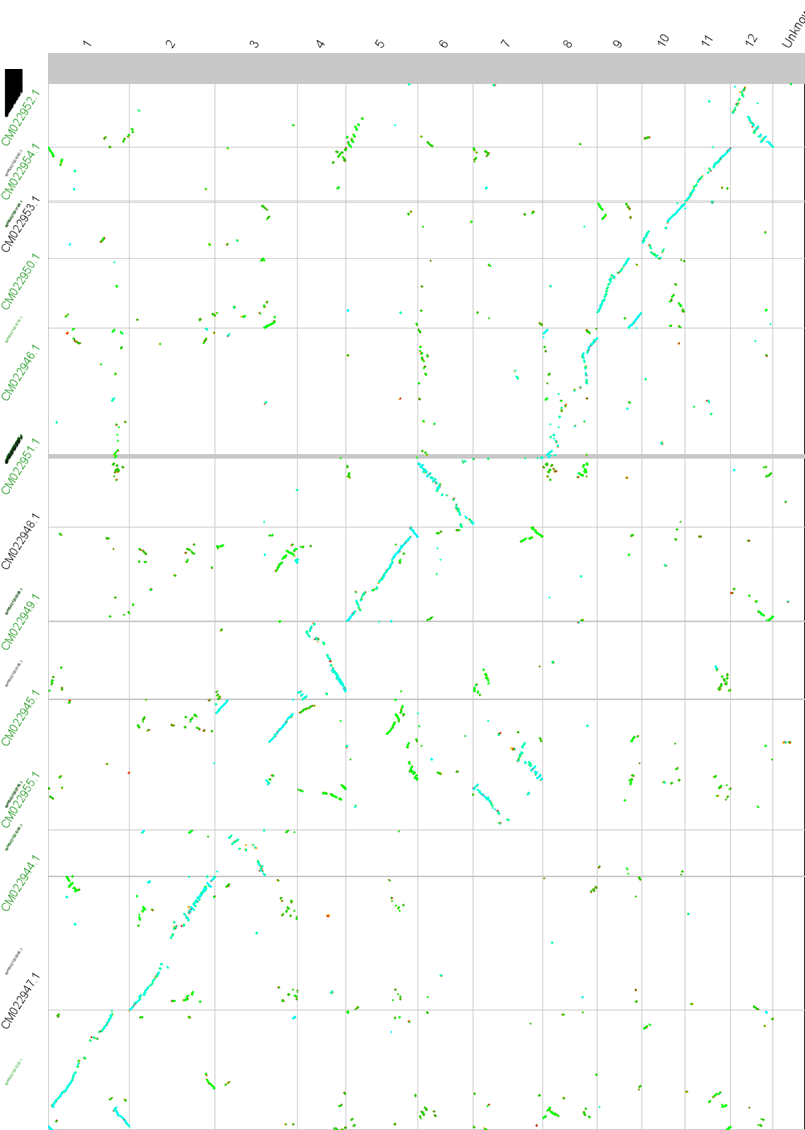


Figure S7: Synteny map of avocado pseudochromosomes against *Litsea cubeba* contigs indicates the closeness of the two species with major parts of the chromosomes conserved between the species.


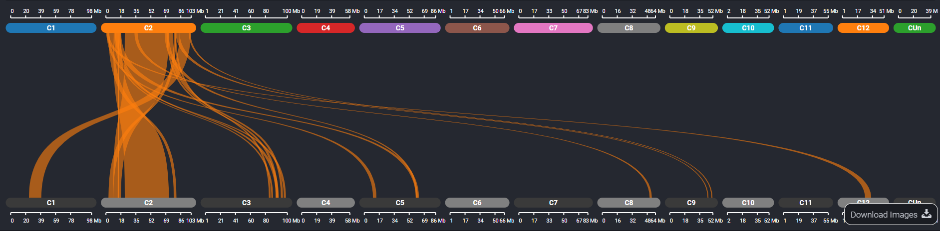

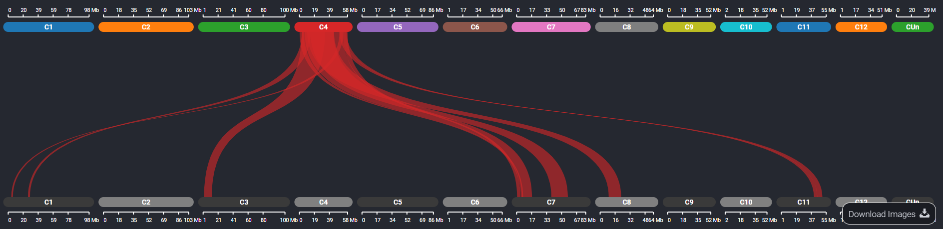

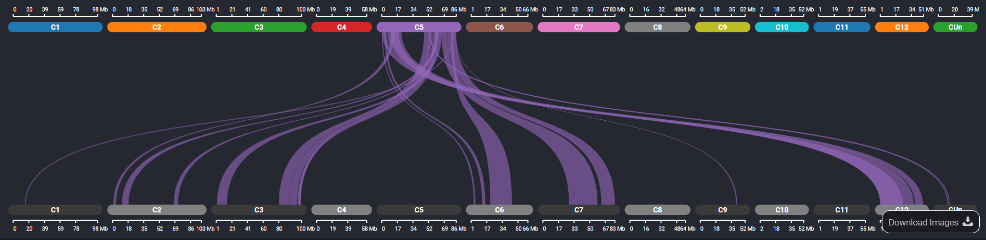

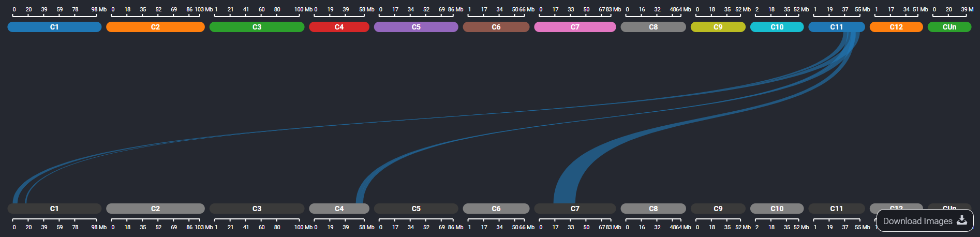

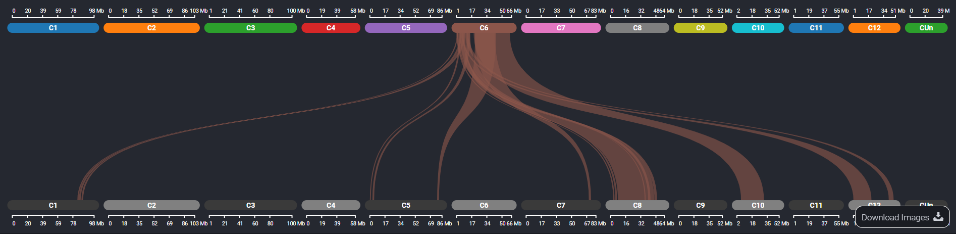

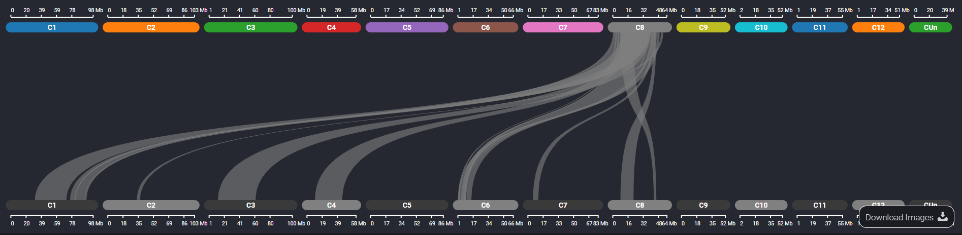

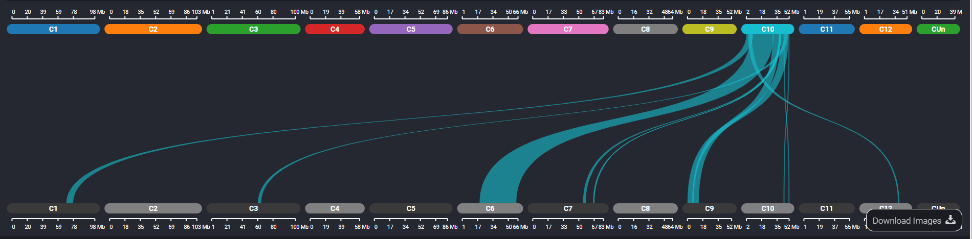

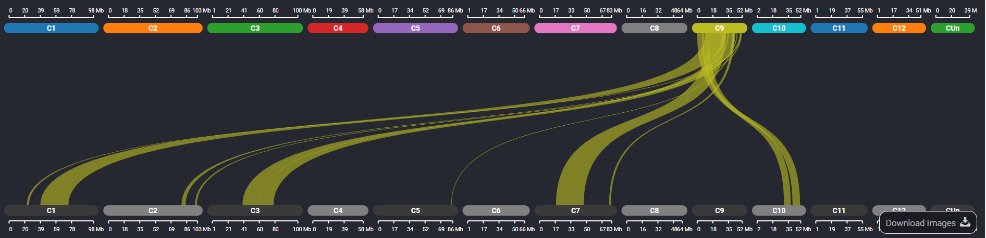

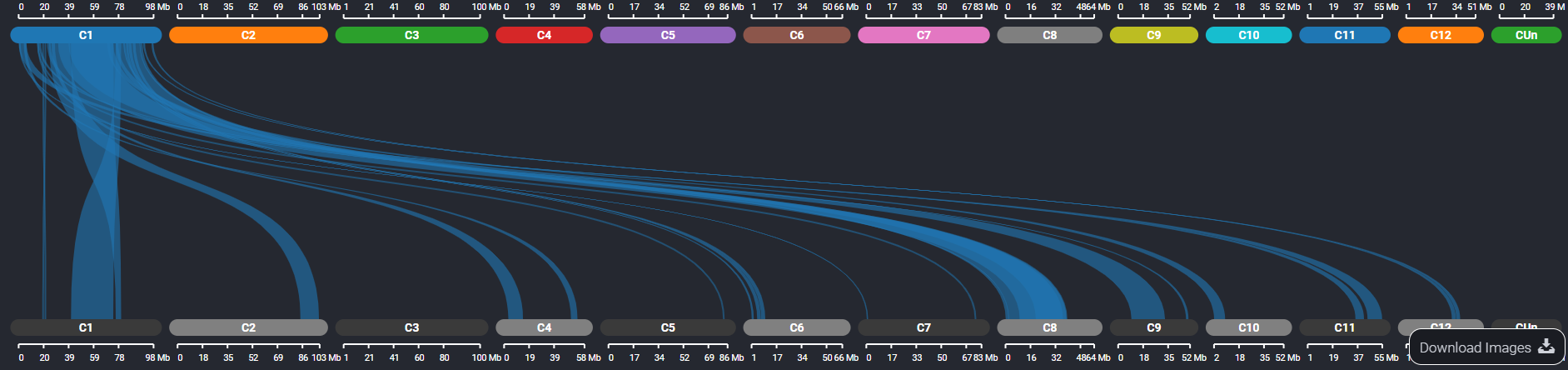

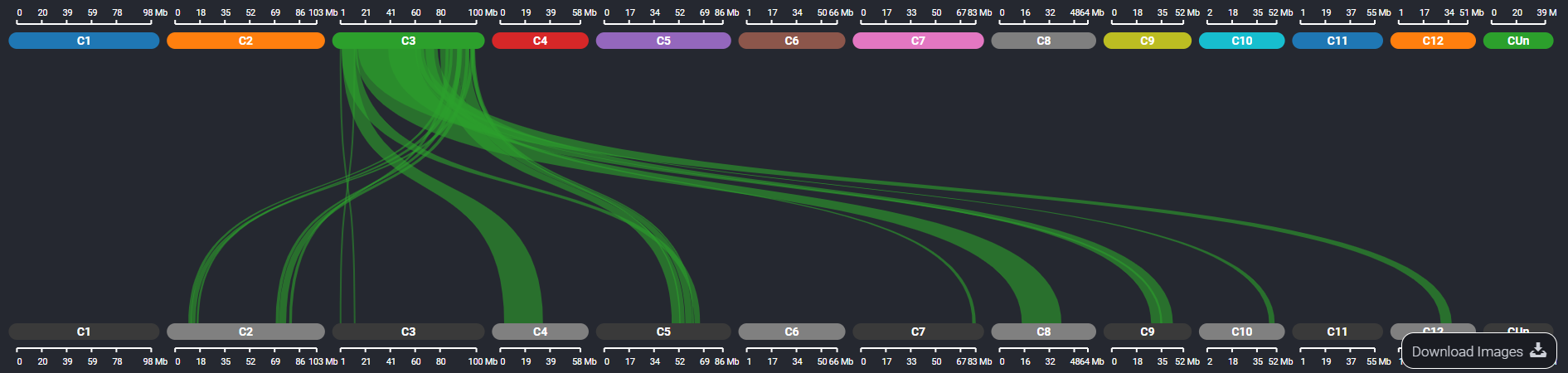


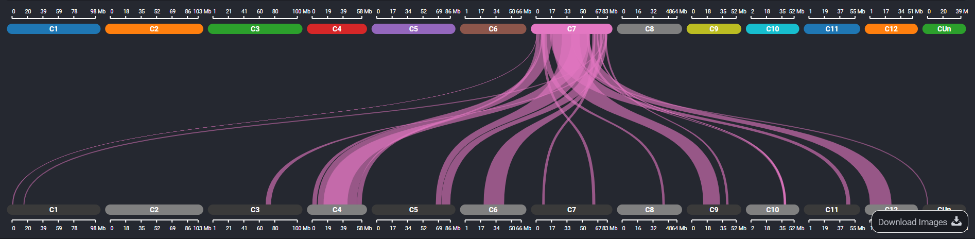


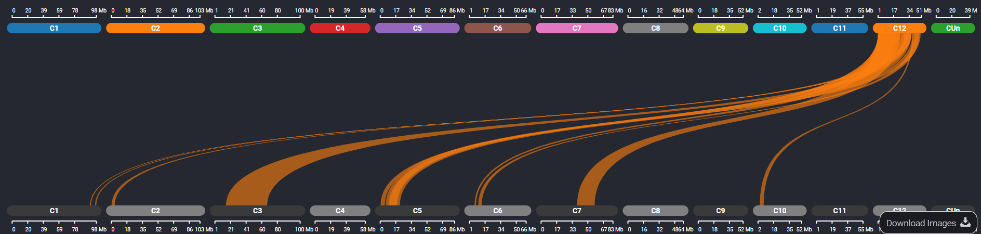


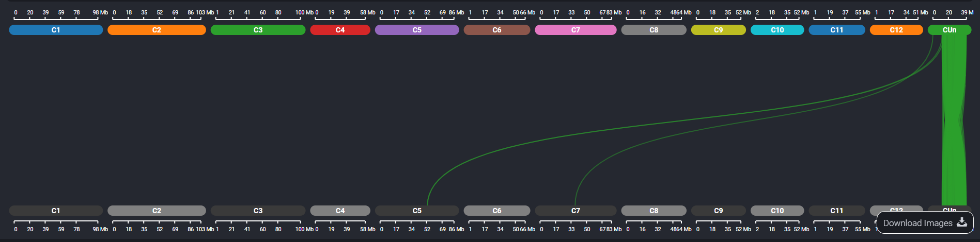


Figure S8: Chromosome wise self-synteny of avocado.

Table S4: Copy number of genes related to Lipid metabolism pathway.

| Gene | Count |
| --- | --- |
| Acyl carrier protein 1 | 3 |
| Acyl carrier protein 2 | 2 |
| Acyl carrier protein 3 | 2 |
| Acyl carrier protein 4 | 1 |
| Acyl carrier protein 5 | 2 |
| Beta-ketoacyl-ACP reductase-like protein | 2 |
| Dihydrolipoyllysine-residue acetyltransferase component 1 of pyruvate dehydrogenase complex | 1 |
| Dihydrolipoyllysine-residue acetyltransferase component 2 of pyruvate dehydrogenase complex | 2 |
| Dihydrolipoyllysine-residue acetyltransferase component 3 of pyruvate dehydrogenase complex | 2 |
| Dihydrolipoyllysine-residue acetyltransferase component 4 of pyruvate dehydrogenase complex | 2 |
| Dihydrolipoyllysine-residue acetyltransferase component 5 of pyruvate dehydrogenase complex | 2 |
| Stearoyl-[acyl-carrier-protein] 9-desaturase | 0 |
| 3-oxoacyl-[acyl-carrier-protein] synthase I | 0 |


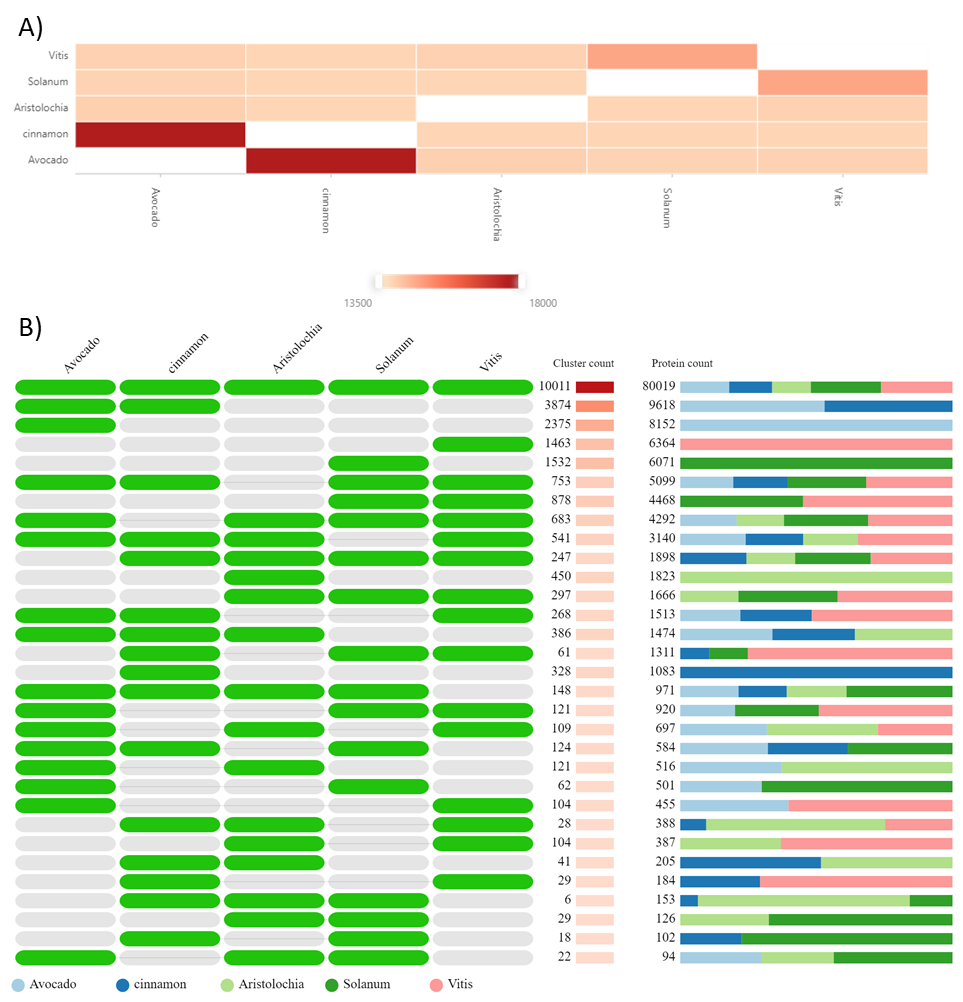


Figure S9: Proteome clustering of 5 genomes. A) Heatmap indicating closeness of species based on clustered proteomes. B) Clusters categorised based on genome combinations, corresponding number of sub-clusters and the stacked bar graph indication ratio of protein sequences from selected species.

**Table S5: Normalised expression of 7C genes in Hass avocado fruit.** Expression is reported as transcripts per kilobase million (TPM). Paired-end RNA-seq read files were obtained from the NCBI Sequence Read Archive and expression data were calculated using Kallisto (https://pachterlab.github.io/kallisto/)

| **Family** | **Gene** | **Mesocarp tissue 110 days after full bloom (SRR9026412) TPM** | **Mesocarp tissue 110 days after full bloom (SRR9026405) TPM** | **Mesocarp tissue 110 days after full bloom (SRR9026406) TPM** | **Mesocarp tissue 110 days after full bloom (SRR9026411) TPM** | **Fruit (SRR9595483) TPM** |
| --- | --- | --- | --- | --- | --- | --- |
| Fructokinase | 27012_t | 11.8293 | 61.4996 | 44.928 | 11.8636 | 23.8425 |
|  | 6329_t | 9.93379 | 5.07437 | 3.49852 | 7.86811 | 0.993257 |
|  | g13119.t1 | 4.34419 | 5.72944 | 4.71577 | 4.59536 | 1.6325 |
|  | g15683.t1 | 8.39228 | 0.339896 | 0.805675 | 29.8613 | 0.416972 |
|  | g2042.t1 | 0.0418179 | 0 | 0 | 0.148359 | 0 |
|  | g21047.t1 | 0.457067 | 0.312957 | 1.11093 | 0.544272 | 0.270897 |
|  | g23015.t1 | 3.37086 | 3.7992 | 4.23424 | 0.996219 | 1.16986 |
|  | g24431.t1 | 0 | 0 | 0 | 0 | 0.0341632 |
|  | g26159.t1 | 0 | 0 | 0 | 0.0905123 | 0 |
|  | g29021.t1 | 0 | 0 | 0 | 0 | 1.53855 |
|  | g29024.t1 | 2.85454 | 2.51035 | 3.23871 | 6.05345 | 1.37289 |
|  | g30005.t1 | 0 | 0 | 0 | 0 | 17.0214 |
|  | g399.t1 | 0.132253 | 1.99082 | 4.07923 | 0 | 1.51389 |
|  | g7201.t1 | 34.3051 | 15.7261 | 11.3949 | 166.023 | 4.03654 |
|  | g8122.t1 | 20.7704 | 47.3651 | 56.4893 | 30.7226 | 31.1718 |
| Fructose-bisphosphate aldolase | 18618_t | 10.4997 | 9.66007 | 20.9006 | 9.41714 | 11.8229 |
|  | 8974_t | 1591.28 | 556.691 | 591.356 | 1378.97 | 463.193 |
|  | g10038.t1 | 0.219476 | 86.6697 | 44.0336 | 0.74599 | 16.9861 |
|  | g15108.t1 | 1574.54 | 642.865 | 701.288 | 1412.62 | 873.312 |
|  | g15552.t1 | 9.46064 | 10.5155 | 11.4847 | 8.73842 | 4.36337 |
|  | g16996.t1 | 4.23703 | 3.4318 | 3.50807 | 3.14956 | 1.30395 |
|  | g25102.t1 | 0 | 0 | 0 | 0 | 0.0746867 |
|  | g26523.t1 | 218.516 | 709.971 | 770.837 | 183.18 | 511.588 |
|  | g29511.t1 | 6.09489 | 9.75053 | 11.4839 | 5.77344 | 4.21398 |
|  | g31463.t1 | 3.101 | 1.89544 | 2.44804 | 2.43753 | 1.21998 |
|  | g3195.t1 | 6.29659 | 3.76494 | 4.48221 | 6.509 | 1.04279 |
|  | g34751.t1 | 4.12984 | 5.07038 | 4.68654 | 4.32833 | 0.902817 |
|  | g34884.t1 | 4.83414 | 12.5891 | 10.0778 | 4.28421 | 2.29656 |
|  | g35114.t1 | 15.853 | 20.9784 | 22.8237 | 15.3369 | 5.79842 |
|  | g41679.t1 | 0 | 0 | 0 | 0 | 0 |
|  | g41680.t1 | 0 | 0 | 0 | 0 | 0 |
|  | g9260.t1 | 335.549 | 586.299 | 585.542 | 385.823 | 616.28 |
|  | g947.t1 | 0 | 0 | 0.0862582 | 0 | 0.929402 |
|  | g948.t1 | 0 | 0.0533081 | 0 | 0 | 27.8409 |
|  | g952.t1 | 1.22792 | 613.416 | 435.115 | 1.47302 | 284.482 |
| Hexokinase | 16945_t | 11.2965 | 4.93338 | 4.38814 | 7.65862 | 0.596766 |
|  | g11066.t1 | 24.3141 | 22.9163 | 22.8632 | 25.7622 | 3.88795 |
|  | g12734.t1 | 0 | 0 | 0 | 0 | 0 |
|  | g13321.t1 | 0 | 0 | 0 | 0 | 0 |
|  | g13323.t1 | 0 | 0 | 0 | 0 | 0 |
|  | g33449.t1 | 4.92541 | 2.25326 | 2.78524 | 5.16249 | 0.856727 |
|  | g36447.t1 | 148.287 | 14.6721 | 18.4948 | 152.71 | 4.76002 |
|  | g36451.t1 | 0 | 0 | 0 | 0 | 0 |
|  | g38840.t1 | 0 | 0 | 0 | 0 | 0 |
|  | g8831.t1 | 36.5082 | 35.6346 | 33.9223 | 23.7437 | 20.5949 |
|  | g999.t1 | 27.5471 | 42.5056 | 51.4766 | 23.817 | 17.4032 |
| Sedoheptulose-1_7-bisphosphatase | 26459_t | 0 | 1.83034 | 0.994535 | 0.132856 | 0 |
|  | g10239.t1 | 0 | 8.9605 | 4.78817 | 0.045643 | 6.60693 |
|  | g10255.t1 | 1.59849 | 8.59522 | 3.27105 | 0.401666 | 4.63757 |
|  | g23332.t1 | 46.5125 | 63.6332 | 68.7763 | 44.5957 | 31.0537 |
|  | g27226.t1 | 0 | 0 | 0 | 0 | 0 |
|  | g35311.t1 | 8.1392 | 20.9646 | 22.1563 | 8.33038 | 14.5303 |
|  | g37724.t1 | 0 | 0 | 0 | 0 | 0 |

**
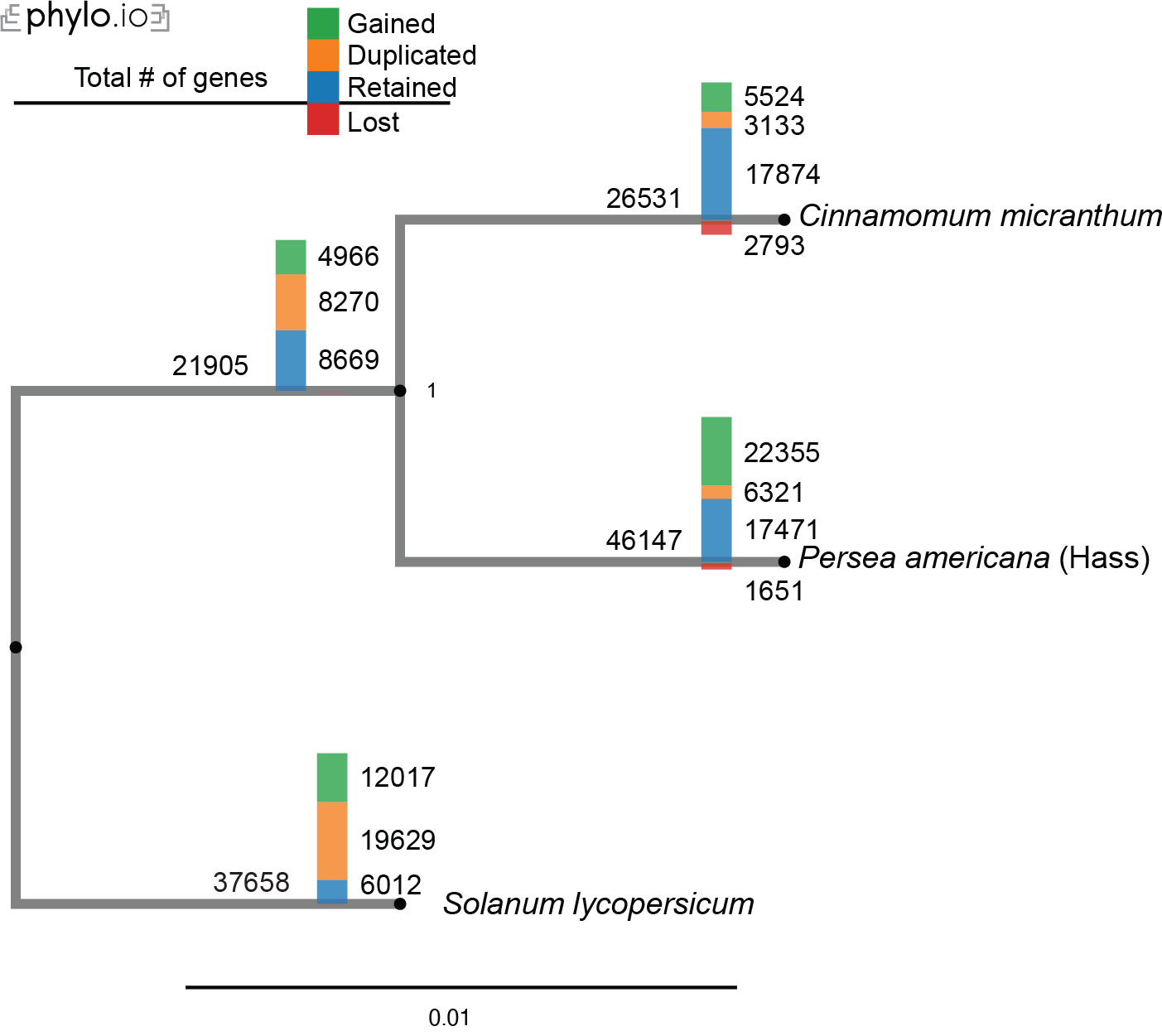
**

Figure S10: Phylogenetic tree of *Persea americiana* (Hass), *Cinnamomum micranthum* and *Solanum lycopersicum*. Labels on the tree show the evolutionary retention, duplication, gain and loss of genes compared to reconstructed ancestral genomes. This analysis was conducted with OMA standalone (<https://omabrowser.org/standalone/>) and visualised using Pyham (<https://lab.dessimoz.org/pyham>).


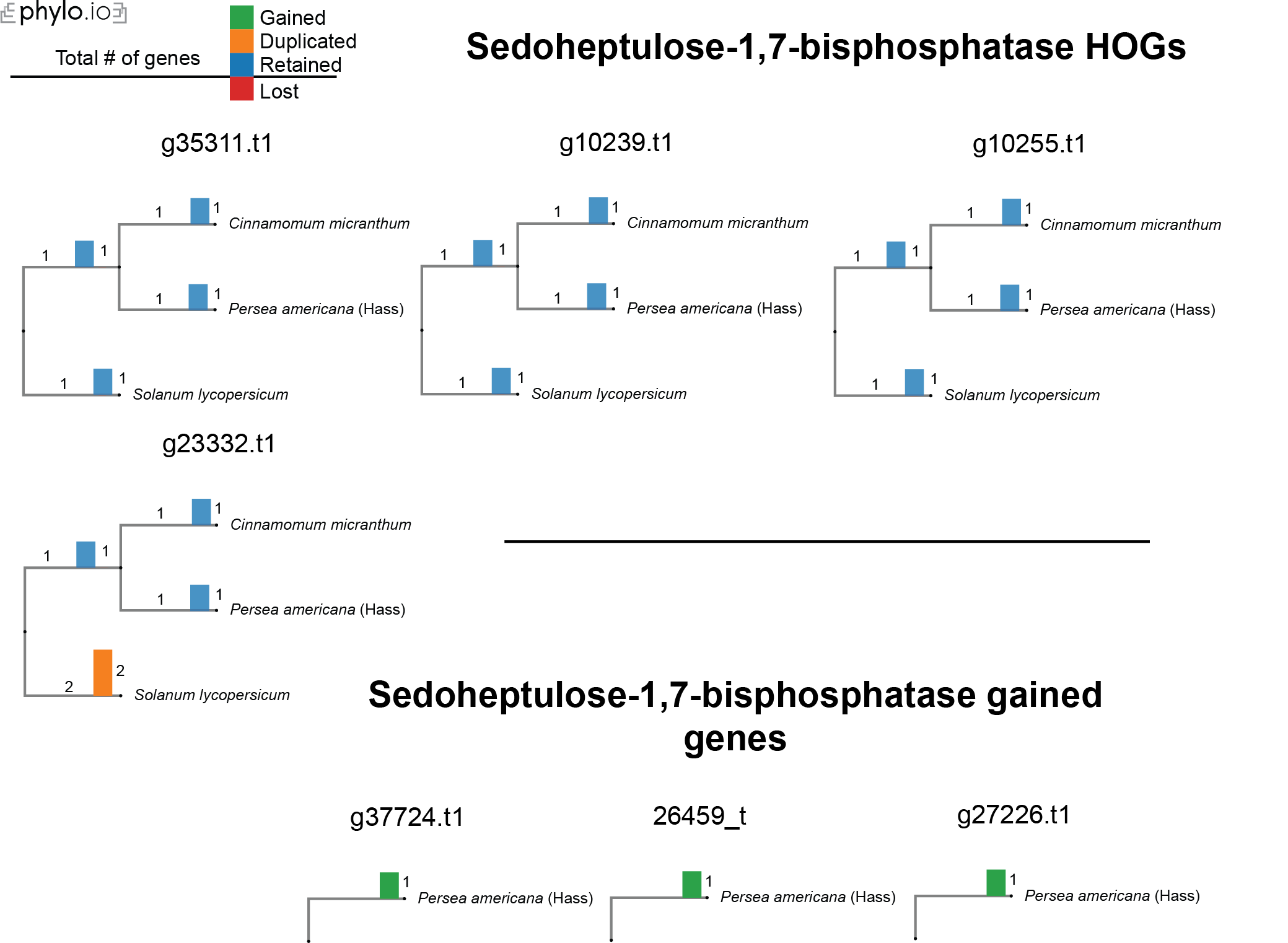


**Figure S11: Sedoheptulose-1,7-biphosphatase Hierarchical Orthologous Groups (HOGs) and gained genes among *Persea americiana* (Hass), *Cinnamomum micranthum* and *Solanum lycopersicum***. Labels on the tree show the evolutionary retention, duplication, gain and loss of genes. Gained genes are singletons in *Persea americania* only. This analysis was conducted with OMA standalone (<https://omabrowser.org/standalone/>) and visualised using Pyham (<https://lab.dessimoz.org/pyham>).


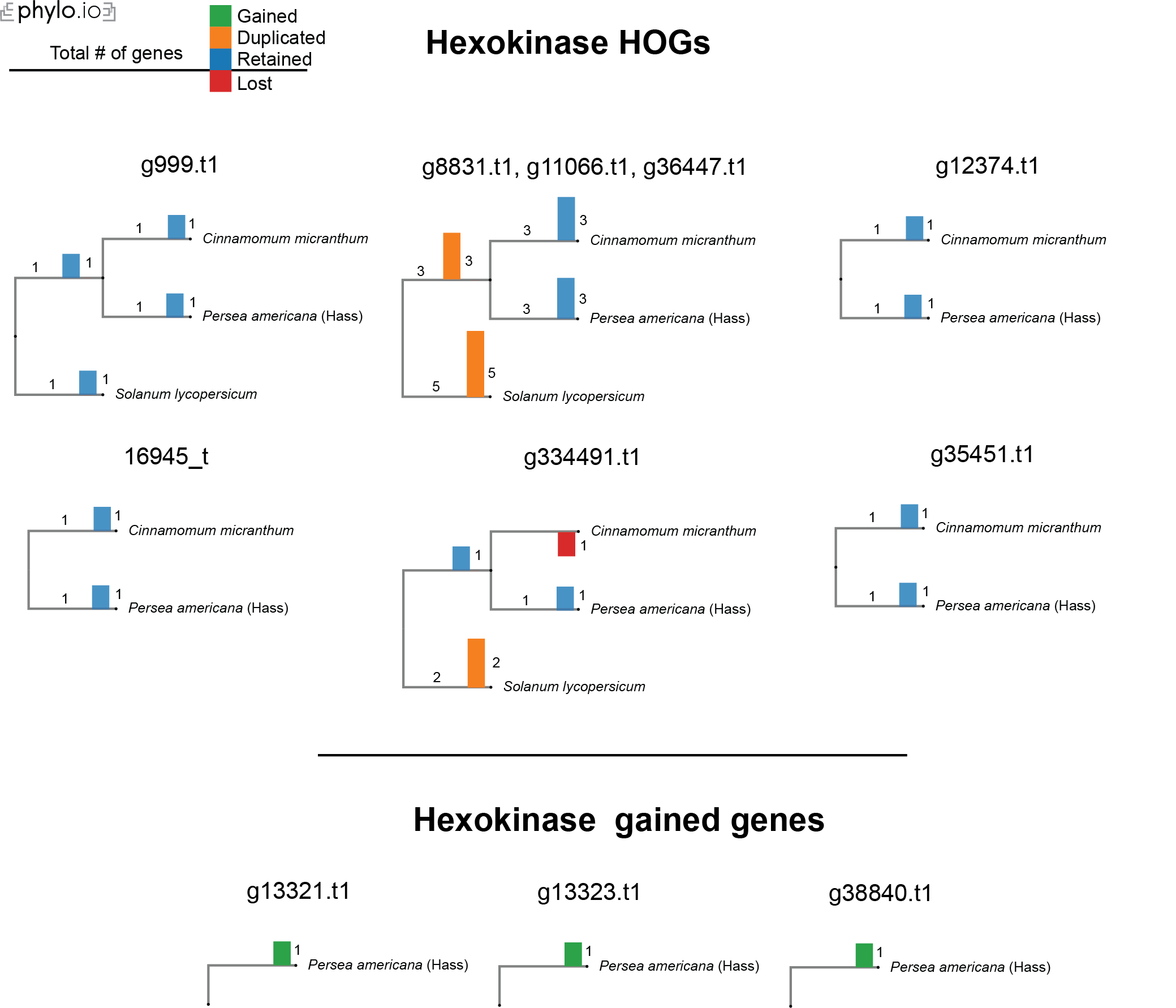


**Figure S12: Hexokinase HOGs and gained genes among *Persea americiana* (Hass), *Cinnamomum micranthum* and *Solanum lycopersicum***. Labels on the tree show the evolutionary retention, duplication, gain and loss of genes. Gained genes are singletons in *Persea americania* only. This analysis was conducted with OMA standalone (<https://omabrowser.org/standalone/>) and visualised using Pyham (<https://lab.dessimoz.org/pyham>).


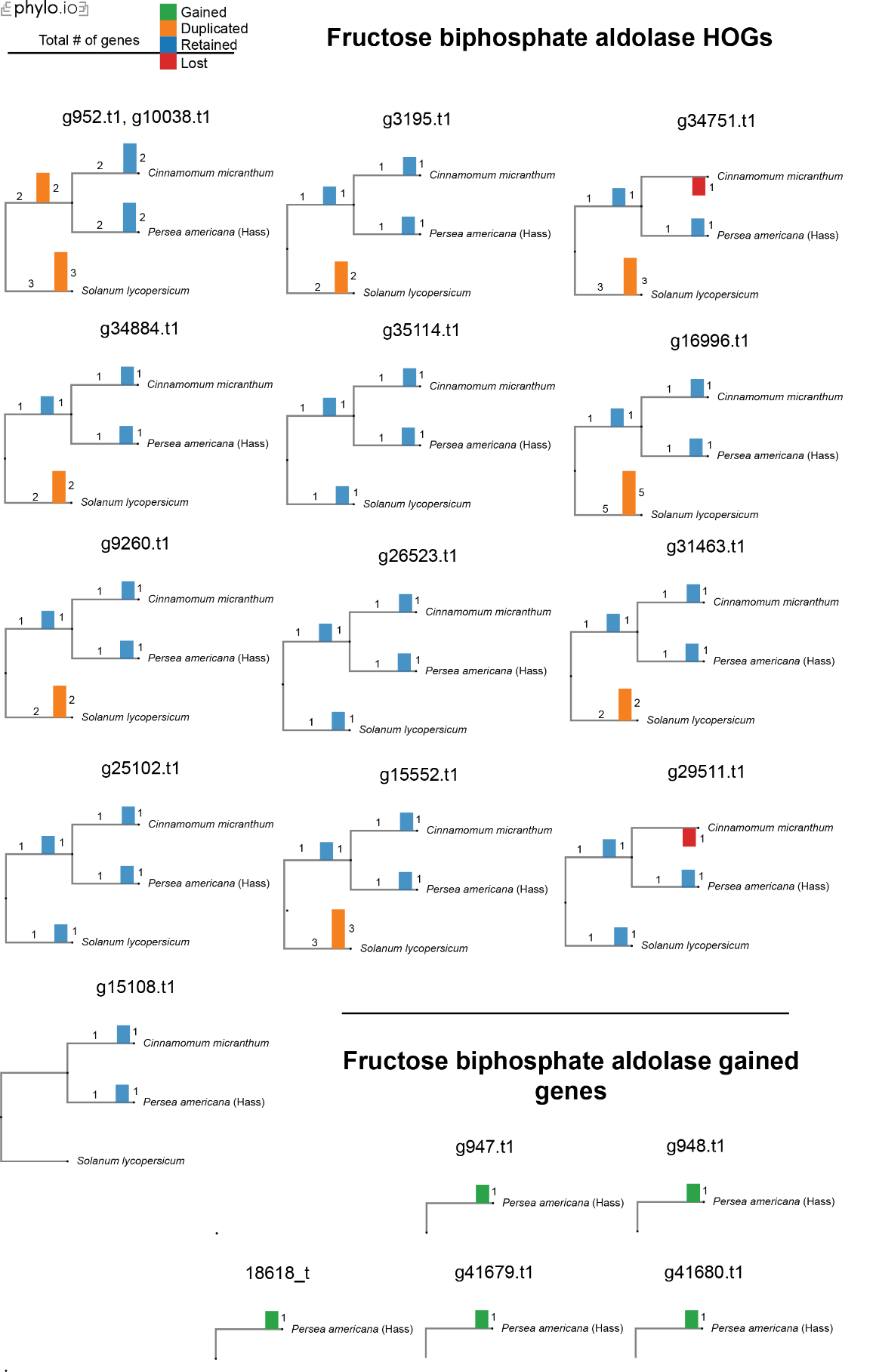


**Figure S13: Fructose biphosphate HOGs and gained genes among *Persea americiana* (Hass), *Cinnamomum micranthum* and *Solanum lycopersicum***. Labels on the tree show the evolutionary retention, duplication, gain and loss of genes. Gained genes are singletons in *Persea americania* only. This analysis was conducted with OMA standalone (<https://omabrowser.org/standalone/>) and visualised using Pyham (<https://lab.dessimoz.org/pyham>).


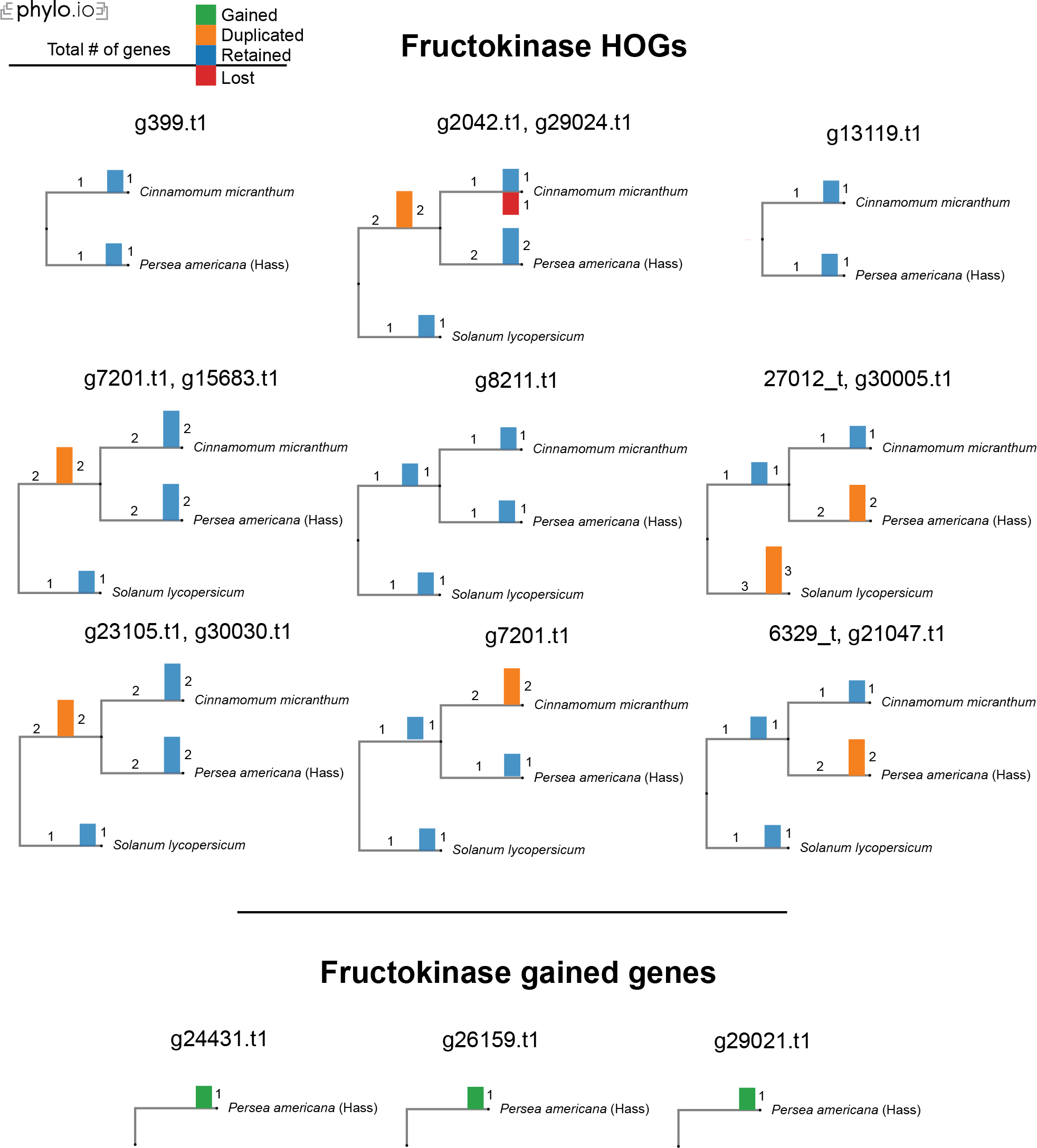


**Figure S14: Fructokinase HOGs and gained genes among *Persea americiana* (Hass), *Cinnamomum micranthum* and *Solanum lycopersicum***. Labels on the tree show the evolutionary retention, duplication, gain and loss of genes. Gained genes are singletons in *Persea americania* only. This analysis was conducted with OMA standalone (<https://omabrowser.org/standalone/>) and visualised using Pyham (<https://lab.dessimoz.org/pyham>).


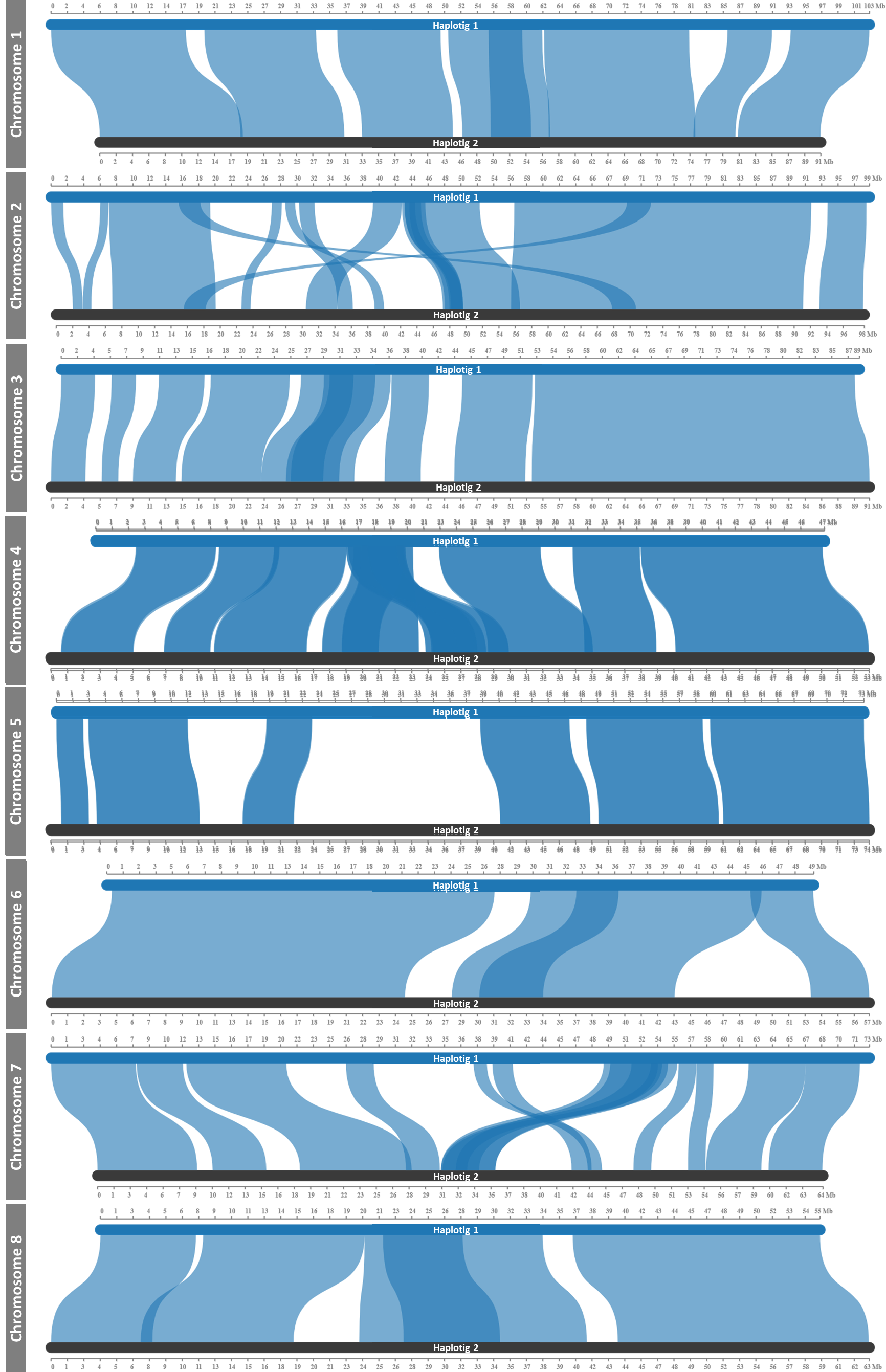

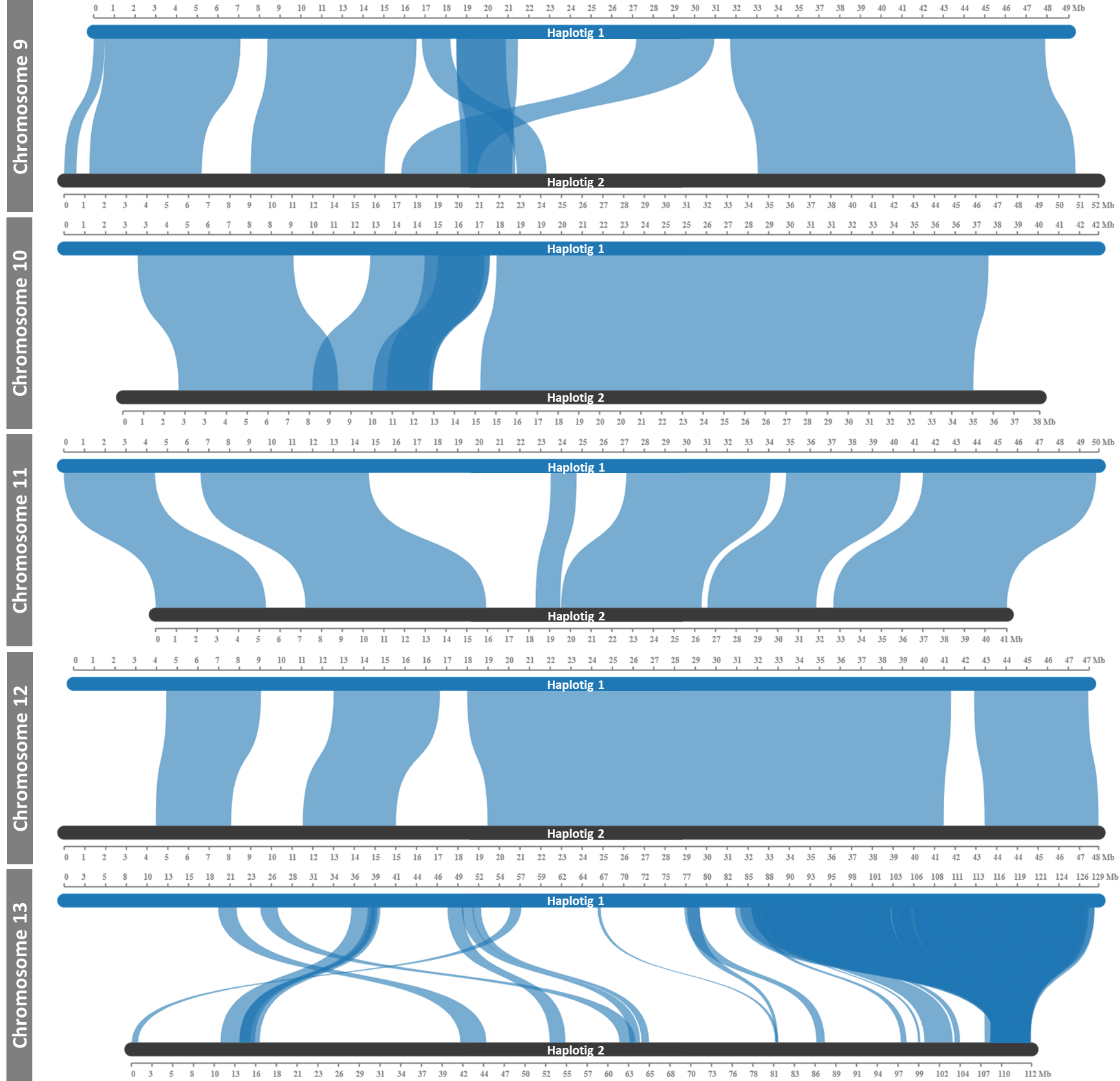


**Figure S15: Comparison maps for the two sets of haplotigs.** Highly conserved regions are indicated by blue connecting segments whereas the regions with sequence differences, insertions and deletions remain unlinked. An e-value cut-off of 1e-50 was used to identify conserved regions.
